# Effect of Delayed Centrifugation on the Levels of NMR-Measured Lipoproteins and Metabolites in Plasma and Serum Samples

**DOI:** 10.1021/acs.analchem.2c02167

**Published:** 2022-12-01

**Authors:** Julia Debik, Sylvia Hetlelid Isaksen, Magnus Strømmen, Manfred Spraul, Hartmut Schäfer, Tone F. Bathen, Guro F. Giskeødegård

**Affiliations:** † Department of Circulation and Medical Imaging, Norwegian University of Science and Technology, Trondheim 7491, Norway; ‡ K.G. Jebsen Center for Genetic Epidemiology, Department of Public Health and Nursing, Norwegian University of Science and Technology, Trondheim 7491, Norway; § Faculty of Medicine and Health Sciences, Norwegian University of Science and Technology, Trondheim 7491, Norway; ∥ Centre for Obesity Research, Clinic of Surgery, St. Olavs Hospital, Trondheim University Hospital, Trondheim 7030, Norway; ⊥ The Clinical Research Ward, Department for Research and Development, St. Olavs Hospital, Trondheim University Hospital, Trondheim 7030, Norway; ## Department of Clinical and Molecular Medicine, Norwegian University of Science and Technology, Trondheim 7491, Norway; ∇ Bruker BioSpin AIC Division, Ettlingen, Rheinstetten 76287, Germany; ○ Department of Radiology and Nuclear Medicine, St. Olavs Hospital, Trondheim University Hospital, Trondheim 7030, Norway; ◆ Clinic of Surgery, St. Olavs Hospital, Trondheim University Hospital, Trondheim 7030, Norway

## Abstract

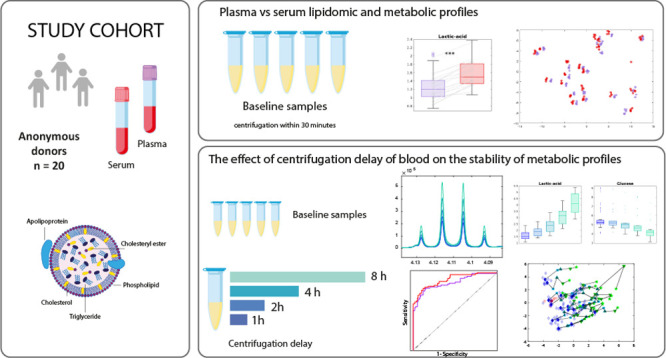

Metabolic profiling is widely used for large-scale association studies, based on biobank material. The main obstacle to the translation of metabolomic findings into clinical application is the lack of standardization, making validation in independent cohorts challenging. One reason for this is sensitivity of metabolites to preanalytical conditions. We present a systematic investigation of the effect of delayed centrifugation on the levels of NMR-measured metabolites and lipoproteins in serum and plasma samples. Blood was collected from 20 anonymous donors, of which 10 were recruited from an obesity clinic. Samples were stored at room temperature until centrifugation after 30 min, 1, 2, 4, or 8 h, which is within a realistic time scenario in clinical practice. The effect of delaying centrifugation on plasma and serum metabolic concentrations, and on concentrations of lipoprotein subfractions, was investigated. Our results show that lipoproteins are only minimally affected by a delay in centrifugation while metabolite levels are more sensitive to a delay. Metabolites significantly increased or decreased in concentration depending on delay duration. Further, we describe differences in the stability of serum and plasma, showing that plasma is more stable for metabolites, while lipoprotein subfractions are equally stable for both types of matrices.

## Introduction

Metabolic profiling is widely used for large-scale epidemiological studies for the search of biomarkers for early disease onset or disease severity.^1−3^ These studies are commonly based on biobank material, in which samples have been collected over years or even decades.^4−6^ There is a large variability in procedures for sample collection, storage, and handling not only between different biobanks but also within the same biobank, especially if the material has been collected in waves or comes from external sources. Metabolites may be affected by different preanalytical factors, and precise information on storage and sample handling is often unavailable.^7−11^ Biomarkers of clinical value need to be verified across different population cohorts, giving rise to an increased interest in international cohort collaborations.^12−14^ Knowledge of how preanalytical steps influence the metabolic profile is important, and tools to assess the quality of existing material would be valuable to exclude or adjust samples with a high deviation from established protocols. Importantly, protocols of new sample collections should be standardized with the aim to reduce preanalytical variability to a minimum.

One of the main analytical methods for metabolic profiling is proton nuclear magnetic resonance (NMR) spectroscopy, which is high throughput, has high reproducibility, and requires minor sample preparation.^15−17^ Low-molecular metabolites from a wide range of chemical classes may be quantified.^16,18,19^ In addition, NMR provides detailed information on lipoproteins.^19−21^ Lipoproteins are lipid carriers consisting of a hydrophobic core made up of triglycerides and cholesterol, surrounded by a monolayer membrane.^22^ Different lipoprotein classes have been defined, based on their density, size, lipid composition, and apolipoproteins, and NMR provides information on lipid and apolipoprotein concentrations, particle numbers, and sizes of several lipoprotein subfractions.^20,21,23^

Previous studies have explored the effect of precentrifugation delay on the stability of some commonly assessed NMR-measured metabolites from blood samples collected in epidemiological studies, showing that in particular metabolites involved in glycolysis are highly affected.^10−12,24−26^ The lactate/glucose concentration ratio has been proposed as a quality assessment tool to identify protocol deviations.^25^ However, there is a need for a systematic evaluation of a broader metabolic panel and lipoprotein subfractions, also including individuals with potentially more extreme lipid values.

Plasma and serum samples are the most common biological materials available in biobanks. Plasma is collected in tubes containing anticoagulant (such as EDTA), which causes the blood to sediment, and thereby separation of blood cells from the whole blood. Serum is collected in tubes without additives, and to obtain serum from whole blood, these tubes must be kept at room temperature for 30–60 min for clotting. The clot contains blood cells, fibrinogen, and clotting factors, which are thus not present in serum. Previous studies have shown that the different collection procedures and coagulation cascades may influence the concentrations of metabolites in the two matrices;^27−30^ however, to the best of our knowledge, the effect of preanalytical conditions on the concentrations of lipoprotein subfractions has not been previously investigated.

The aim of this study was to investigate the effect of delayed centrifugation on the stability of NMR-measured metabolites and lipoproteins in plasma and serum samples within a realistic time scenario in clinical practice. To assess metabolite and lipoprotein stability within a broad range of blood lipid values, we included both healthy volunteers and participants under follow-up at an obesity clinic. Additionally, differences in metabolic profiles of plasma and serum samples were investigated to determine if measurements of metabolites and lipoprotein subfractions differed in the two biological matrices.

## Materials and Methods

### Sample Collection and Experimental Design

Blood samples were obtained from 20 anonymous donors, of which 10 were recruited from the obesity clinic at St. Olavs Hospital, Trondheim University Hospital. Plasma samples were collected in EDTA-plasma vacuette tubes, while serum was collected in serum vacutainer tubes with a clot activator, and all samples were divided into nine aliquots. Samples were kept at the bench at room temperature (RT) until centrifugation after 30 min (5 aliquots), 1, 2, 4, or 8 h (1 aliquot each). Samples were stored at −80 °C until NMR analysis. After thawing at RT for approximately 30 min, 300 μL of plasma or serum was mixed with 300 μL of buffer [D_2_O (20% in H_2_O) with 0.075 M Na_2_HPO_4_, 6 mM NaN_3_, 4.6 mM 3-(trimethylsilyl)-2,2,3,3-tetradeuteropropanoic acid (TSP-d_4_), pH 7.4] and transferred to 5 mm NMR tubes. Quality control (QC) samples, prepared from pooled samples (six samples in total), were run in parallel to assess the quality of the NMR acquisitions and identify instrumental drifts. NMR analyses were carried out on a Bruker Avance III HD Ultrashield Plus 600 MHz spectrometer (Bruker BioSpin) equipped with a 5 mm TCI probe. Sample handling and data acquisition were automatically performed using our standard in-house protocol (details in the Supporting Material). NMR spectra were recorded using one-dimensional nuclear Overhauser effect spectroscopy (1D-NOESY) and Carr–Purcell–Meiboom–Gill (CPMG) experiments. The success of the NMR experiments was assessed based on shim quality (e.g., line width of the alanine doublet at ∼1.5 ppm < 1.5 Hz incl. line broadening), size of the residual water signal (e.g., its concentration equivalent < 30 mmol/L), and TSP peak (28.1–43.7 mmol/L), and samples that did not meet the quality requirements were prepared from left-over material of the corresponding aliquot. This study, using anonymized samples, was classified as a quality control study by the Regional Committee for Medical and Health Research Ethics in Central Norway. Ethical approval was thus not required to conduct this study, but it was approved by and performed according to existing regulations for quality control studies at the Clinic of Surgery, St. Olavs Hospital, Trondheim University Hospital.

### Metabolite Quantification

Forty-one metabolites were automatically quantified using the B.I. Quant-PS 2.0 software (Bruker BioSpin) from plasma and serum samples. This software is based on an algorithm developed for fitting predefined proton signals from the 1D-NOESY spectra. Metabolites with concentrations below the limit of detection (LOD) for more than 30% of the samples were disregarded to avoid biased results from a high fraction of imputed values, and thus 33 plasma and 32 serum metabolites were retained for statistical analysis. None of the removed metabolites had concentrations lower than LOD for fewer than 30% of the baseline samples. Values below LOD were imputed using an expectation-maximization algorithm from the zComposition package in R.^31^

### Lipoprotein Parameter Analysis

Lipoprotein subfractions were automatically quantified using Bruker IVDr Lipoprotein Subclass Analysis (B.I.LISA) software from Bruker BioSpin, which provides a detailed picture of circulating lipoproteins.^19^ Concentrations of lipids [cholesterol (CH), free cholesterol (FC), triglycerides (TG), and phospholipids (PL)] in total plasma/serum and in 4 main lipoprotein classes, very low, intermediate-, low-, and high-density lipoproteins (VLDL, IDL, LDL, and HDL), and 15 subclasses (VLDL 1–5, LDL 1–6, and HDL-1–4) are provided by the software. In addition, levels of apolipoproteins (Apo-A1, Apo-A2, and Apo-B) in the lipoproteins, 12 calculated parameters (ratios of LDL-CH/HDL-CH and Apo- B/Apo-A1), and particle numbers of total VLDL, IDL, LDL, and LDL 1–6 are provided, giving a total of 112 lipoprotein subfractions. Calculated parameters and particle numbers were excluded from the statistical analysis, i.e., 100 lipoprotein subfractions were utilized for further analysis. Values equal to zero were present in 12 and 20 lipoprotein subfractions in plasma and serum samples, respectively. The proportion of zero-values made up less than 10% for all subfractions, and zero-values were imputed similarly to the metabolites.

### Statistical Analysis

To visually assess variation in the metabolic profiles within and between donors, principal component analysis (PCA) was carried out in Matlab R2021a (The MathWorks Inc.) using PLS Toolbox 8.7.1.^32,33^ By connecting samples from the same individual in PCA trajectory plots, the presence or absence of systematic changes was observed. In addition, univariate analysis of log-transformed metabolite and lipoprotein concentrations was performed with linear mixed models (LMMs), including centrifugation delay as a fixed effect (continuous variable) and individual ID as a random effect. Correct model assumptions were confirmed by QQ-plots of the residuals. In addition, coefficients of variations (CVs) were calculated from the baseline samples (five samples of 30 min centrifugation delay) and from all samples of an individual (the mean of the baseline samples, 1, 2, 4, and 8 h centrifugation delays) to compare the analytical variability with the variability as an effect of delayed centrifugation. Percentage changes of metabolic concentrations under a centrifugation delay, as compared to the baseline samples, were also calculated to assess the magnitude of the effect. The glucose/lactate concentration ratio as a tool for the identification of samples with a delayed centrifugation (above 30 min) was assessed by receiver operating characteristics (ROCs) using the pROC package in R.^34,35^

Wilcoxon signed-rank tests were used to assess differences in concentrations of quantified metabolites and lipoprotein subfractions measured in plasma and in serum samples, using the mean concentrations of each individual from baseline samples only (30 min centrifugation delay). *P*-values were corrected for multiple testing using the Benjamini–Hochberg procedure, and significance was considered for *Q*-values ≤ 0.05.^36^

## Results

### Reproducibility of Quantified Metabolites and Lipoprotein Subfractions

CVs of metabolites, calculated from plasma QC samples, were below 15% and below 20% for 23 and 27 of the metabolites, respectively (Table S1). CVs of metabolites of serum QC samples were below 15% for 22 metabolites and below 20% for 25 metabolites. All lipoprotein subfractions, in both matrices, had CVs below 10% (Table S2).

### Effect of Delayed Centrifugation on Serum and Plasma Metabolites

**Figure 1:**
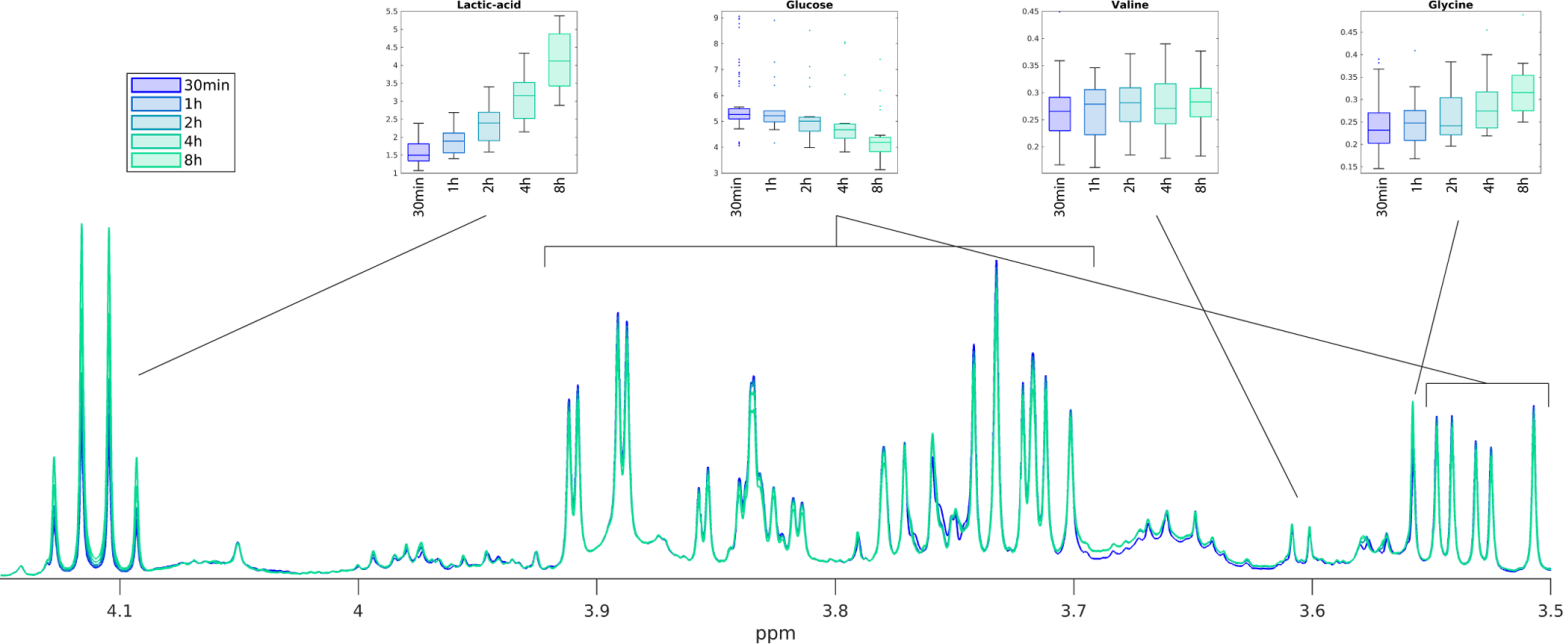
Mean raw CPMG serum spectra for samples that have been centrifuged within 30 min (blue), 1, 2, 4, and 8 h (turquoise).

Figure 1 shows the mean raw NMR spectra of serum samples with different centrifugation delays, clearly showing that several metabolites are highly affected even by a 1 h centrifugation delay. Some metabolites decreased in concentration (e.g., glucose), others increased (e.g., lactate, glycine), while some were minimally affected (e.g., valine) by a delay. In plasma spectra (Figure S1), we observed a high increase of the K-EDTA peaks between 30 min and 1 h centrifugation delay, which was saturated within 1 h.

**Figure 2:**
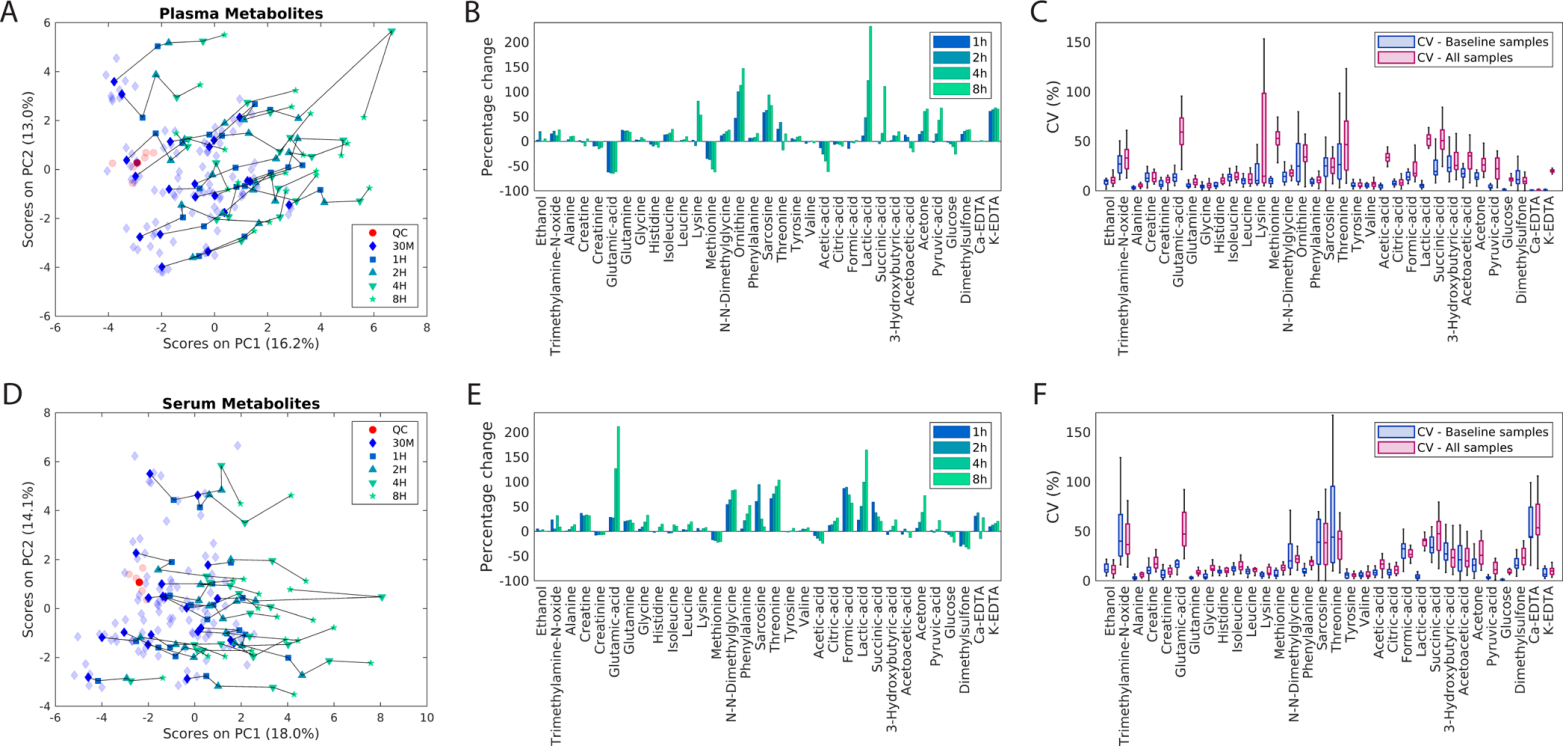
(A + D) PCA trajectory plots of plasma (A) and serum (D) metabolic profiles in samples with different centrifugation delays (30 min, 1, 2, 4, and 8 h). The length of the delay is indicated by a distinct color and marker type. Samples from the same individual are connected by a line. Baseline samples are indicated by transparent markers, and their average is indicated by a blue diamond. Similarly, QC samples are indicated by transparent red circles, while their average is indicated by a solid red circle. One major outlier may be seen in panel (A) and two in panel (D). Spectra corresponding to these samples met the quality control requirements of the quantification algorithms, and thus they were not removed from statistical analyses. (B + E) Average percentage changes of plasma (B) and serum (E) metabolite levels when centrifugation has been delayed for 1–8 h, compared to the baseline levels. (C + F) CVs of plasma (C) and serum (F) metabolites calculated from baseline samples only (blue) compared to including all samples (pink). The baseline samples were averaged in the later. PCA: principal component analysis; PC: principal component; CV: coefficient of variation.

Figure 2A,D shows PCA score plots for quantified plasma and serum metabolites, respectively, where samples are colored according to the length of the centrifugation delay, demonstrating that the largest variation in the data (along the first principal component, PC1) is due to a centrifugation delay. The corresponding loading plots for PC1 can be found in Figures S2 and S3, showing the main changes in metabolite levels due to centrifugation delays. Twenty-five of 33 plasma metabolites and 25 of 32 serum metabolites had a significant change in concentration (Table S3). Percentage changes in metabolite concentrations for plasma and serum metabolites compared to the baseline samples exceeded ±50% for some metabolites already within a 1 h centrifugation delay (Figure 2B,E and Table S4). Metabolites with the highest percentage decrease in concentration include methionine (−61 and −20% decrease within an 8 h delay in plasma and serum, respectively), acetic acid (−61 and −24%), and glucose (−25 and −21%). Metabolites with the highest increase in concentrations after an 8 h delay were lactic acid (+232 and +164% increase for plasma and serum, respectively), ornithine (+147% in plasma, excluded in serum), and *N*-*N*-dimethylglycine (+23 and +84% increase). Interestingly, the glutamic acid concentration had a significant decrease in plasma (−61%), while it increased in serum (+212%), and the concentration of dimethyl-sulfone significantly increased in plasma (+24%), while it decreased in serum (−35%). CVs calculated from baseline samples compared with those calculated when including samples with a centrifugation delay show that delaying centrifugation adds a substantial amount of extra variability to the data (Figure 2C,F and Table S5). Mean CVs calculated from baseline samples were 13.1 and 17.7% for plasma and serum samples, respectively, while they increased to 23.6 and 22.2% including all samples (including averaged baseline levels).

### Lactate/Glucose Ratio as a Marker of Delayed Centrifugation

The area under the receiver operating curve (ROC-AUC) of the lactate/glucose concentration ratio for classifying if a sample as had a centrifugation delay exceeding 30 min was 0.87 and 0.91 for plasma and serum, respectively (Figure S4A). A longer delay (2–8 h) could be identified with a higher accuracy (AUC = 0.94 and 0.95 for plasma and serum, respectively). The lactate/glucose ratio increases linearly with the duration of a centrifugation delay, where the increase is at a higher rate in plasma than in serum (Figure S4B).

### Effect of Delayed Centrifugation on Lipoprotein Subfractions

**Figure 3:**
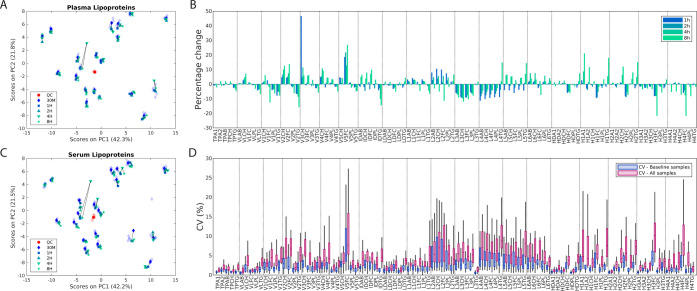
PCA trajectory plots of plasma (A) and serum (C) lipoprotein subfractions in samples with different centrifugation delays (30 min, 1, 2, 4, and 8 h). The length of the delay is indicated by a distinct color and marker type. Samples from the same individual are connected by a line. Baseline samples are indicated by transparent markers, and their average is indicated by a blue diamond. Similarly, QC samples are indicated by transparent red circles, while their average is indicated by a solid red circle. One major outlier may be seen in panel (A) and two in panel (C). Spectra corresponding to these samples met the quality control requirements of the quantification algorithms, and thus they were not removed from statistical analyses. (B) Average percentage changes of plasma levels of lipoprotein subfractions when centrifugation has been delayed for 1–8 h, compared to the baseline levels. (D) CVs of plasma lipoprotein subfractions calculated from baseline samples only (blue) compared to including all samples (pink). The baseline samples were averaged in the later. PCA: principal component analysis; PC: principal component; CV: coefficient of variation; TP: total plasma; VLDL: very low density lipoprotein; IDL: intermediate-density lipoprotein; LDL: low-density lipoprotein; HDL: high-density lipoprotein; CH: cholesterol; FC: free cholesterol; PL: phospholipids; TG: triglycerides. AB: apolipoprotein-B; A1: apolipoprotein-A1; A2: apolipoprotein-A2.

**Figure 4:**
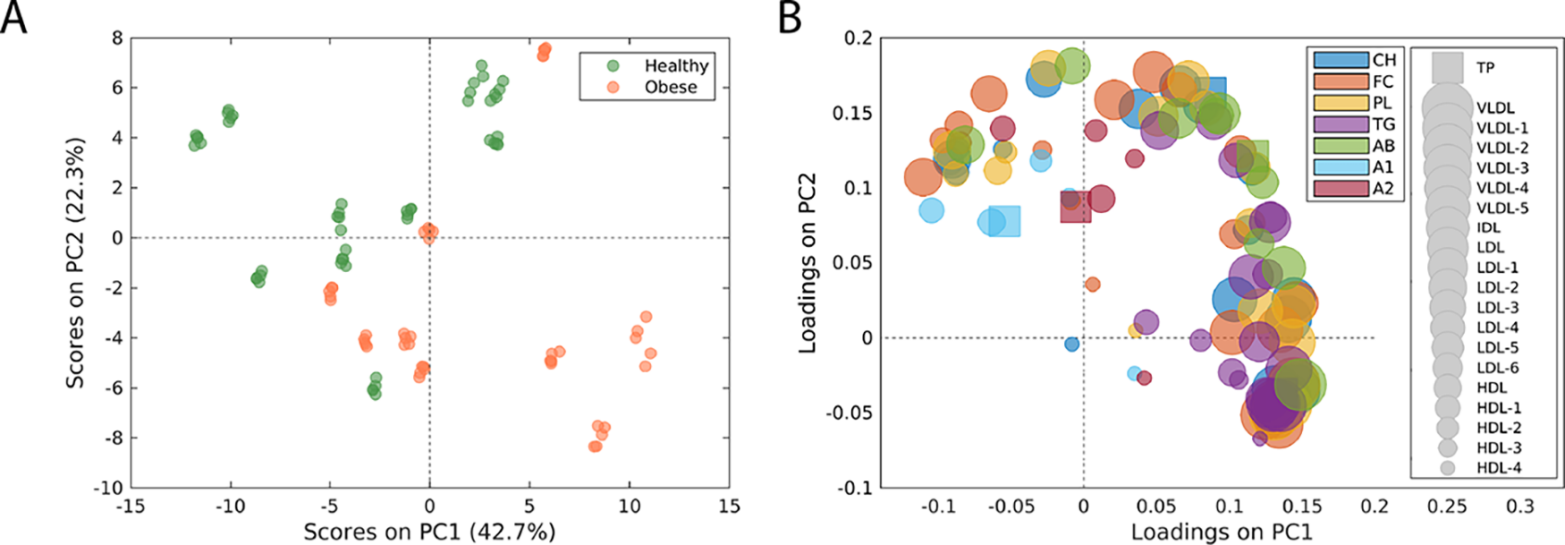
(A) PCA score plot of levels of plasma lipoprotein subfractions in baseline sample aliquots of healthy volunteers (green) and individuals included from the obesity clinic (orange). (B) Loadings on PC1 and PC2 comparing the lipoprotein profiles of healthy volunteers and individuals included from the obesity clinic. Different subfractions are represented by different sizes of the points in the figure, and the properties of the subfractions are represented by different colors. Squared points indicate total plasma concentrations, while circles indicate lipoprotein subfractions. PCA: principal component analysis; PC: principal component; TP: total plasma; VLDL: very low density lipoprotein; IDL: intermediate-density lipoprotein; LDL: low-density lipoprotein; HDL: high-density lipoprotein; CH: cholesterol; FC: free cholesterol; PL: phospholipids; TG: triglycerides, AB: apolipoprotein-B; A1: apolipoprotein-A1; A2: apolipoprotein-A2.

For lipoprotein subfractions, between-individual variation was substantially higher than variation caused by delaying centrifugation for both plasma and serum, as samples from the same individuals cluster closely together (Figures 3A,C, S5, and S6). Seventy-eight lipoprotein subfractions measured in plasma and 74 in serum had a significant change in concentration (Table S6). However, although significant, the percentage changes in concentrations were low. For plasma, percentage differences were below ±20% within an 8 h delay for all but four variables (Figure 4B and Table S7). Importantly, average changes in concentrations were in general not systematic. Variables with the highest percentage decrease were free cholesterol in HDL-4 and HDL-3 particles, with a −21.6 and −21.5% average decrease, respectively. Variables with the highest increase were apo-A1 concentrations in HDL-1 and free cholesterol in VLDL-5, with average increases of +20.9 and +26.8%, respectively. When quantified from serum, the percentage changes were slightly higher (Figure S7 and Table S7), with nine variables exceeding ±20% for an 8 h delay. Greatest decreases in concentrations were observed within the LDL-2 subfraction, with average decreases of −42.6, −39.2, −24.8, and −30% for free cholesterol, cholesterol, apo-B, and phospholipids, respectively. Concentrations of triglycerides in LDL-4 and LDL-5 and free cholesterol and cholesterol in main LDL had the greatest increases, which were +34.0, +30.6, +33.6, and +62.4%, respectively. Figure 4D shows CVs of lipoprotein subfractions quantified from plasma calculated from baseline samples, compared to those including all samples, showing that lipoproteins overall have a very high analytical reproducibility, and even with an added extra variability due to a delay in centrifugation, the CVs are mostly beneath 10% (Table S8). A corresponding figure for serum lipoproteins can be found in Figure S8. Mean CVs calculated from baseline samples were 2.7% for plasma and 3.3% for serum samples and increased to 5.2 and 6.3% when including all samples, respectively. The highest increases in CVs were observed for serum levels of apo-B, cholesterol, free cholesterol, and phospholipids in LDL-2, with an increase in CVs in the range of 12–29%.

### Effect of Obesity on the Stability of Plasma and Serum Metabolic Profiles

Scores and loading plots from PCA of lipoprotein subfractions quantified from baseline plasma aliquots show that lipid profiles of participants from the obesity clinic are characterized by significantly elevated levels of triglycerides and VLDLs and decreased levels of HDLs (Figures 4A,B, S9, and S10 and Table S9). Importantly, the stability of lipoprotein subfractions under a delayed centrifugation is not affected by the concentration levels (Figures S11 and S12). Plasma CVs of lipoprotein subfractions measured from participants from the obesity clinic (mean CV = 2.7%) are comparable to CVs of the healthy volunteers (mean CV = 2.8%) and in general low. Equivalent figures may be found for serum lipoprotein subfractions in Figures S13 and S14. Serum CVs of participants from the obesity clinic had a mean value of 3.8%, while the mean CV for healthy volunteers was 2.7%.

Scores and loading plots from PCA on metabolite concentrations of baseline plasma aliquots (Figures S13–S16) also show that the metabolic profiles differ slightly in these two groups of participants. Obese participants were characterized by significantly increased plasma levels of creatinine, isoleucine, valine, and glucose and decreased glutamine, glycine, histidine, methionine, and acetic acid levels (Table S10). Metabolic alterations caused by a centrifugation delay were not dependent on the obesity status (Figures S17 and S18). The mean CV of plasma metabolites from participants from the obesity clinic was 23.0%, while the mean CV for healthy volunteers was 24.2%. Similar results were found for serum metabolic profiles (Figures S12–S14), where participants from the obesity clinic had a mean CV of 22.8%, while that of healthy volunteers was 21.6%.

### Differences between Plasma and Serum Metabolites and Lipoprotein Subfractions

**Figure 5:**
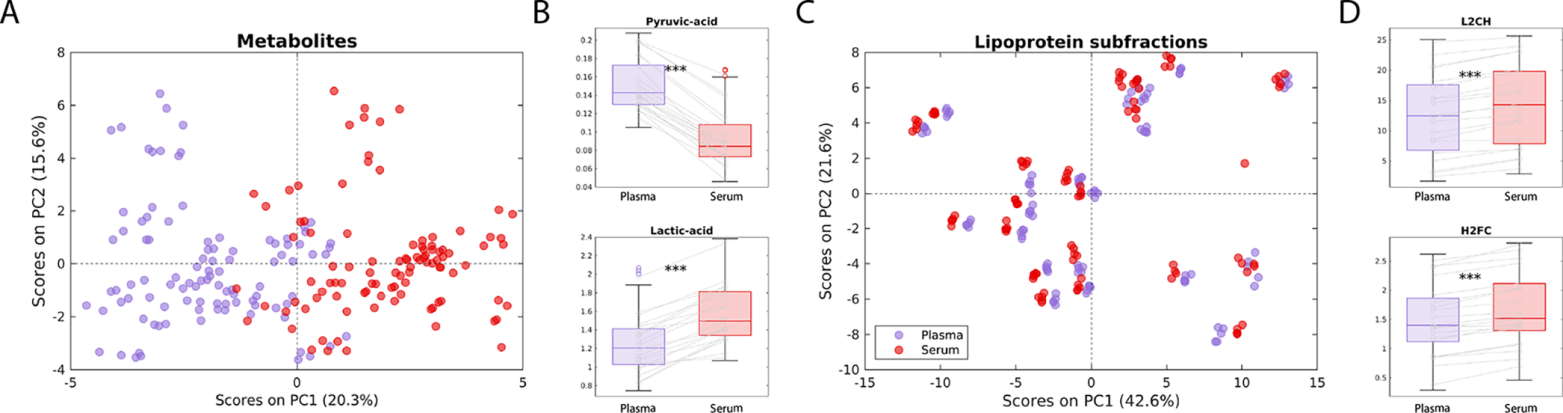
(A) PCA score plots of metabolite levels quantified from plasma (purple) and serum (red) baseline samples. (B) Boxplots showing the difference in metabolite levels in plasma and serum baseline samples for pyruvic acid and lactic acid. (C) PCA score plots of lipoprotein subfractions quantified from plasma and serum baseline samples. (D) Boxplots showing the difference in the levels of lipoprotein subfractions L2CH and H2FC in plasma and serum baseline samples. Gray lines connect measurements from the same individuals. ^***^Multiple-correction adjusted *P*-values from Wilcoxon signed-rank test <0.01. PCA: principal component analysis; PC: principal component; TPTG: total plasma triglycerides; L2CH: LDL-2 cholesterol; H2FC: HDL-2 free cholesterol.

Figure 5A shows the score plots from PCA performed on metabolites quantified from plasma and serum baseline samples, with the exclusion of EDTA concentrations. A clear difference in metabolic profiles of serum and plasma was observed, exemplified by the boxplots showing metabolic concentrations for two chosen metabolites (Figures 5B and S19). Furthermore, Wilcoxon signed-rank tests showed that out of 32 metabolites quantified both in serum and in plasma, 28 had significantly different concentrations in the two types of biological matrices, after correcting for multiple testing (Table S11).

Metabolites with the largest average percentage increase in serum concentration levels, relative to plasma, were creatinine (400% increase), leucine (189%), and methionine (110%), while metabolites with the largest average decrease were pyruvic acid (40% decrease), glutamic acid (67%), and acetic acid (33%).

Differences in concentrations were substantially smaller for lipoprotein subfractions, as visualized in Figures 5C and S20. PCA score plots show that the interindividual variation is much higher than variation caused by quantification from a different matrix. Samples from the same individuals cluster closely together, and the boxplots in Figure 5D show that even though some concentrations are significantly different, the differences in concentrations are small. Wilcoxon signed-rank tests showed that out of 100 lipoprotein subfractions, 91 had significantly different concentrations after correcting for multiple testing (Table S12). Average percentage differences in serum levels of lipoproteins relative to plasma levels were below ±20% for all but five lipoprotein subfractions.

## Discussion

Blood is the most collected biological matrix for large-scale epidemiology studies utilizing metabolomics for the search of new potential biomarkers for early disease onset or disease severity. In this work, we have systematically investigated the effect of a delay in centrifugation (1, 2, 4, and 8 h) on the levels of NMR-measured metabolites and lipoprotein subfractions in a sample population with a wide range of lipid values.

### Lipoprotein Subfractions Are Minimally Affected by a Delay in Centrifugation, also in Obese Patients

We show that lipoprotein subfractions are only minimally affected by a delay in centrifugation. This is in accordance with a previous study, which evaluated the effect of centrifugation delays of 24 and 48 h on the stability of lipoprotein subfractions.^12^ The additional value of our study is the inclusion of individuals from an obesity clinic, allowing an investigation of the stability of lipoproteins from participants with a wide range of lipid profiles. Our results demonstrated that lipoprotein subfractions are highly reproducible regardless of the specific lipid profile.

In a previous study in which we investigated the effect of repeated freeze and thaw cycles on NMR-measured metabolites and lipoproteins, we showed that lipoproteins are substantially less affected than metabolites, indicating, in general, a higher robustness to preanalytical sample handling.^8^ The performance criteria of the National Cholesterol Education Program (NCEP) Laboratory Standardization Panel for lipoprotein testing need to be met by any analytical technique used for patient lipoprotein assessment.^37−40^ The performance criteria for total cholesterol, total triglycerides, LDL-cholesterol, and HDL-cholesterol are CV <3.0, <5, <4.0, and <4.0%, respectively. In this study, we demonstrate that measurements of these variables in samples with a delay in centrifugation up to 8 h, stored at room temperature, comply with the NCEP performance criteria, both when measured in plasma and serum. The CVs including all samples were for total cholesterol 1.5% in plasma and 1.8% in serum; for total triglycerides 2.0% in plasma and 1.9% in serum; for LDL-cholesterol 2.2% in plasma and 3.5% in serum; and for HDL-cholesterol 1.6% in plasma and 2.2% in serum (Table S6).

### Metabolites are Vulnerable to Delayed Centrifugation

In contrast to lipoproteins, metabolites were affected by a delayed centrifugation (centrifugation after 1 h or more). Lactate concentrations increased more than 150% after an 8 h delay, while glucose decreased more than 20% in both matrices. Although several studies have similarly investigated the effect on the stability of NMR metabolic profiles, most studies to date have been performed on NMR spectra and not quantified metabolites and by investigating cohorts of limited sample size.^10,11,24−26,41^ In agreement with our study, increased lactate levels have been reported by others.^10,12,25,26,42^ For example, Fliniaux et al. investigated centrifugation delays of 4 and 24 h on serum samples kept at 4 °C or in room temperature, showing that integrated spectral buckets corresponding to lactate and glucose were highly affected at 25 °C but not for samples kept at 4 °C.^24^ Bervoets et al. reported an increase in lactate and a decrease in pyruvate and glucose, with centrifugation delays of 3 and 8 h at 4 °C in serum from six individuals.^26^ In contrast to this, pyruvate levels were increased in both serum and plasma in our study. Bernini et al. similarly investigated both serum and plasma samples when delaying the centrifugation for 1–4 h, at 4 and 25 °C, concluding that degradation processes are time-dependent and temperature-dependent for both serum and plasma samples and that incubation at 25 °C causes larger changes to the NMR profile.^10^

In addition to previously reported metabolites altered by a prolonged centrifugation delay, we found other metabolites highly affected. For example, plasma ornithine levels increased by 147% when compared to the baseline levels. Other metabolites highly affected were phenylalanine (15.0 and 52.5% increase in plasma and serum, respectively) and alanine (10.4 and 13.7% increase). Serum glycine increased by 32.9%, while plasma levels increased by 4.5%. The region 2.5–2.5 ppm was highly affected by an increase in the spectral baseline, thus affecting metabolites in that region, e.g., pyruvate and 3-hydroxybutyric acid (Figure S21). A similar baseline distortion was not observed in plasma samples.

### Lactate/Glucose Ratio as a Measure for Compliance with the Analytical Protocol

Changes in lactate and glucose concentrations by delayed centrifugation are mainly driven by red blood cell activity, as the same metabolites have been shown to be minimally affected by poststorage conditions (buffer addition delay and NMR profiling delay).^12^ Our results show that changes in concentrations occurred already with a centrifugation delay of 1 h and further increased during the 8 h delay. The lactate/glucose concentration ratio has been suggested as an indicator of samples with delayed centrifugation.^25^ We confirmed that the ratio could separate samples according to centrifugation delay with high accuracy and showed that the ratio increases linearly with increased delay. Further, we show that the ratio increases at a higher rate in plasma compared to that in serum, which is in accordance with the study by Jobard et al.^25^ Storing samples at 4 °C, after the necessary 30 min at RT needed for clotting, has shown to keep metabolite levels much more stable.^10−12,24,25^ The ratio does, however, have a high interindividual variability and may be affected by external factors such as diet, time since the last meal, physical activity levels, and diseases.^43−48^ Nevertheless, it may be used to identify samples with a high deviation from an analytical protocol where this information is lacking. A multivariate metabolic signature has been proposed to determine compliance with standard procedures and quality assessment of blood samples.^49^ We, however, argue that including a broader metabolic panel is less convenient as different metabolites may be present or quantifiable in different cohorts.

### Differences in Metabolic and Lipid Profiles of Plasma and Serum Samples

Comparing plasma and serum metabolic profiles showed that metabolites are present in different concentrations in the two different types of biological matrices. This is in accordance with previous studies comparing metabolic concentrations in blood matrices.^27,29,30,50^ The choice of sample material may thus greatly affect the results in biomarker studies, and this should be taken into consideration when comparing results across studies with different blood matrices. Overall, plasma showed the highest reproducibility for most of our measured metabolites with respect to centrifugation delay, compared to serum. Based on QC samples, plasma metabolites had the highest reproducibility with CVs <25% for all but two metabolites (threonine and glutamic acid) and although the spectra are dominated by large EDTA peaks, investigating the raw spectra showed that these peaks did not distort neighboring metabolite peaks. In contrast, five metabolites (3-hydroxybutyric acid, formic acid, sarcosine, succinic acid, and trimethylamine-*N*-oxide) had CVs > 25% in serum. Percentage changes in metabolite levels caused by delaying centrifugation were on average highest for serum metabolites; however, some metabolites (e.g., lactic acid) were more affected in plasma. Concentrations of lipoprotein subfractions were much more robust under a delayed centrifugation. Similarly, as for metabolites, lipoprotein subfractions measured in plasma had smaller percentage differences on average compared to serum. Collectively, for studies with different blood matrices, metabolite results should be compared with caution, while lipoprotein subfractions can be compared more directly.

## Conclusions

This study has investigated the effect of a delayed centrifugation (1–8 h) on the concentrations of NMR-measured small-molecular metabolites and lipoprotein subfractions in plasma and serum samples. Metabolic profiles were clearly affected by a centrifugation delay already after 1 h, in particular metabolites involved in anaerobic glycolysis. Processing delay should thus be as short as possible; however, this is often difficult to achieve in practice in a clinical setting. In general, plasma was more robust to delay in centrifugation compared to serum; however, some metabolites were more stable in serum. Only small variations were observed for concentrations of lipoprotein subfractions, which demonstrated a higher resilience to preanalytical handling. Our results pinpoint the need for standardization of sample handling across labs and biobanks to make results across different cohorts comparable, with a minimization of time until centrifugation and storage at low temperatures if possible.

## Supporting Information Available

Tables with coefficients of variation; mean raw plasma spectra; PCA scores and loading plots; results from linear mixed models; mean percentage changes in concentrations of metabolites/lipoproteins under a centrifugation delay; mean metabolite/lipoprotein levels of individuals included in the obesity clinic and healthy volunteers; mean metabolite concentrations in plasma and serum samples; and baseline distortion of spectra (PDF)
