# Supplementary material for: Effect of Delayed Centrifugation on the Levels of NMR-Measured Lipoproteins and Metabolites in Plasma and Serum Samples
Source: Anal Chem. 2022 Dec 1;94(49):17003–10. doi: 10.1021/acs.analchem.2c02167 (PMC9753045; doi:10.1021/acs.analchem.2c02167)
Supplement: Supplementary file 1 — ac2c02167_si_001.pdf [file ac2c02167_si_001.pdf]

Supplementary material

Effect of delayed centrifugation on the levels of NMR-measured lipoproteins and metabolites in plasma and serum samples

Julia Debik[1,2], Sylvia Hetlelid Isaksen[3], Magnus Strømmen[4,5,6] Manfred Spraul[7], Hartmut Schaefer[7], Tone F. Bathen[1,8], Guro F. Giskeødegård[2,9]

[1] *Department of Circulation and Medical Imaging, Norwegian University of Science and Technology, Trondheim, Norway*

[2] *K.G. Jebsen Center for Genetic Epidemiology, Department of Public Health and Nursing, Norwegian University of Science and Technology, Trondheim, Norway*

[3] *Faculty of Medicine and Health Sciences, Norwegian University of Science and Technology, Norway*

[4] *Centre for Obesity Research, Clinic of Surgery, St. Olavs Hospital, Trondheim University Hospital, Trondheim, Norway*

[5] *The Clinical Research Ward, Department for Research and Development, St. Olavs Hospital, Trondheim University Hospital, Trondheim, Norway*

[6] *Department of Clinical and Molecular Medicine, Norwegian University of Science and Technology, Trondheim, Norway*

[7] *Bruker BioSpin AIC Division, Ettlingen, Germany*

[8] *Department of Radiology and Nuclear Medicine, St. Olavs Hospital, Trondheim University Hospital, Trondheim, Norway*

[9] *Clinic of Surgery, St. Olavs Hospital, Trondheim University Hospital, Trondheim, Norway*

Corresponding authors:

Julia Debik: julia.b.debik@ntnu.no

Guro F. Giskeødegård: guro.giskeodegard@ntnu.no

TABLE OF CONTENTS

Details on Sample Collection and Experimental Design

Table S1. Coefficients of variation (CVs) of metabolites quantified in plasma and serum quality control (QC) samples.

Table S2. Coefficients of variation (CVs) of lipoprotein subfractions measured in plasma and serum quality control (QC) samples.

Figure S1. Mean raw plasma spectra for different lengths of a centrifugation delay

Figure S2. PCA loadings plot of plasma metabolites in samples with a different length of centrifugation delay.

Figure S3. PCA loadings plot of serum metabolites in samples with a different length of centrifugation delay

Table S3. Results from linear mixed models (LMMs) for assessing the significance of changes in metabolite concentrations with a delay in centrifugation (1h-8h).

Table S4. Mean percentage changes of metabolite levels under a centrifugation delay of 1h, 2h, 4h and 8h, compared to baseline concentrations (centrifugation within 30 minutes)

Table S5. Coefficients of variation (CV) of metabolite concentrations calculated including only the baseline samples (centrifugation within 30 minutes) and including all samples.

Figure S4. ROC curve for the lactate/glucose ratio.

Figure S5. PCA loadings plot of plasma levels of lipoprotein subfractions in samples with a different length of centrifugation delay.

Figure S6. PCA loadings plot of serum levels of lipoprotein subfractions in samples with a different length of centrifugation delay.

Table S6. Results from linear mixed models (LMMs) for assessing the significance of changes in lipoprotein subfraction concentrations with a delay in centrifugation (1h-8h).

Table S7. Mean percentage changes of levels of lipoprotein subfractios under a centrifugation delay of 1h, 2h, 4h and 8h, compared to baseline concentrations (centrifugation within 30 minutes)

Figure S7. Percentage changes of serum levels of lipoprotein subfractions.

Table S8. Coefficients of variation (CV) of concentrations of lipoprotein subfractions calculated including only the baseline samples (centrifugation within 30 minutes) and including all samples.

Figure S8. CVs of lipoprotein subfractions measured in serum samples.

Figure S9. PCA scores plot of serum levels of lipoproteins in healthy vs obese individuals.

Figure S10. PCA loadings plot of serum lipoproteins of healthy vs obese individuals.

Table S9. Mean concentrations of lipoprotein subfractions (in mg/dL) between individuals included into the obesity clinic and healthy volunteers.

Figure S11. CV of plasma levels of lipoprotein subfractions in healthy vs obese individuals.

Figure S12. CVs of serum levels of lipoprotein subfractions in healthy vs obese individuals.

Figure S13. PCA scores plot of plasma metabolites in healthy vs obese individuals.

Figure S14. PCA loadings plot of plasma metabolites in healthy vs obese individuals.

Figure S15. PCA scores plot of serum metabolites in healthy vs obese individuals.

Figure S16. PCA loadings plot of serum metabolites in healthy vs obese individuals.

Table S10. Mean metabolite levels (in mmol/L) between individuals included into the obesity clinic and healthy volunteers.

Figure S17. CVs of plasma metabolites in healthy vs obese individuals.

Figure S18. CVs of serum metabolites in healthy vs obese individuals.

Figure S19. PCA loadings plot of metabolites quantified from plasma vs serum of the same individuals.

Table S11. Differences in metabolite levels measured in plasma and serum samples.

Figure S20. PCA loadings plot of lipoprotein subfractions measured in plasma vs serum samples of the same individuals.

Table S12. Differences in levels of lipoprotein subfractions measured in plasma and serum samples.

Figure S21. Baseline distortion of serum spectra.

Sample Collection and Experimental Design:

Blood samples were obtained from 20 anonymous donors, of which 10 were recruited from the obesity clinic at St. Olavs Hospital, Trondheim University Hospital. Plasma samples were collected into EDTA Plasma vacuette tubes, while samples for serum were collected in serum vacutainer tubes with clot activator, and all samples were divided into 9 aliquots. Samples were kept at the bench in room temperature, until centrifugation after 30 minutes (5 aliquots), 1h, 2h, 4h or 8h (1 aliquot each). Plasma and serum samples were then stored at -80°C until NMR analysis. After thawing at room temperature for approximately 30 minutes, 300 µL plasma or serum was mixed with 300 µL buffer [D2O (20% in H2O) with 0.075 M Na2HPO4, 6 mM NaN3, 4.6 mM 3-(trimethylsilyl)-2,2,3,3-tetradeuteropropanoic acid (TSP-d4), pH 7.4], and transferred to 5mm NMR tubes. Quality control (QC) samples were prepared from pooled samples (6 samples in total). One QC sample was run per day to assess the quality of the NMR acquisitions and identify instrumental drifts. NMR analyses were carried out on a Bruker Avance III HD Ultrashield Plus 600 MHz spectrometer (Bruker BioSpin) equipped with a 5 mm TCI probe. Sample handling and data acquisition were automatically performed using SampleJet sample changer with cooling and the automation software IconNMR on Topspin 3.5 (Bruker BioSpin). NMR spectra were recorded using one dimensional nuclear Overhauser effect spectroscopy (1D-NOESY) and Carr-Pucell-Meiboom-Gill (CPMG) experiments. Both experiments were performed at 310 K and applied presaturation by irradiation (25 Hz) on the water resonance during relaxation delay (4 s) and mixing time (10 ms). The 1D-NOESY experiment ("noesygppr1d", Bruker nomenclature) was performed using 96k data points and 30 ppm spectral width. 32 scans were recorded, and the free induction decays were Fourier-transformed after zero filling (128k real data points) and 0.3 Hz line broadening. The CPMG experiment (pulse sequence "cpmgpr1d", Bruker nomenclature) was recorded with 72k data points, 20 ppm spectral width and 32 scans. Data was zero filled to 128k data points, line broadening (0.3 Hz) before Fourier-transformation. The success of the NMR experiments was assessed based on shim quality (eg. linewidth of the alanine doublet at ~1.5 ppm < 1.5 Hz incl. line broadening), size of residual water signal (e.g. its concentration equivalent < 30 mmol/L), and TSP peak (28.1-43.7 mmol/L), and samples which did not meet the quality requirements were prepared from left-over material of the corresponding aliquot. This study using anonymized samples was classified as a quality control study by the Regional Committee for Medical and Health Research Ethics in Central Norway. An ethical approval was thus not required to conduct this study, but it was approved by and performed according to the regulations for quality control studies at the Clinic of Surgery, St. Olavs Hospital, Trondheim University Hospital.

Table S1. *Coefficients of variation (CVs) of metabolites quantified in plasma and serum quality control (QC) samples.*

**Metabolite**	**CV Plasma (%)**	**CV Serum (%)**
3-Hydroxybutyric-acid	19.9	40.0
Acetic-acid	11.7	10.4
Acetoacetic-acid	9.2	8.2
Acetone	16.4	15.2
Alanine	4.9	3.4
Ca-EDTA	1.2	65.7
Citric-acid	10.1	11.7
Creatine	14.3	13.4
Creatinine	15.6	12.9
Dimethylsulfone	8.2	18.4
Ethanol	12.8	14.7
Formic-acid	20.0	76.0
Glucose	1.2	2.0
Glutamic-acid	25.3	14.5
Glutamine	7.5	3.6
Glycine	4.7	8.1
Histidine	13.0	14.5
Isoleucine	11.2	11.2
K-EDTA	1.4	8.4
Lactic-acid	5.7	8.5
Leucine	14.7	5.0
Lysine	14.1	6.9
Methionine	6.1	4.6
N-N-Dimethylglycine	35.0	36.5
Phenylalanine	6.7	14.3
Pyruvic-acid	3.6	4.7
Sarcosine	7.4	60.7
Succinic-acid	18.8	38.7
Threonine	96.3	15.6
Trimethylamine-N-oxide	24.4	45.1
Tyrosine	5.3	7.2
Valine	9.4	5.8

Table S2. *Coefficients of variation (CVs) of lipoprotein subfractions measured in plasma and serum quality control (QC) samples.*

**Lipoprotein subfraction**		**CV Plasma (%)**	**CV Serum (%)**
Totals	TPA1	0.29	0.29
	TPA2	1.06	1.06
	TPAB	1.46	1.46
	TPCH	0.65	0.65
	TPTG	0.88	0.88
VLDL	VLAB	0.65	0.65
	VLCH	4.34	4.34
	VLFC	1.27	1.27
	VLPL	0.86	0.86
	VLTG	0.58	0.58
VLDL-1	V1CH	3.37	3.37
	V1FC	1.61	1.61
	V1PL	1.32	1.32
	V1TG	1.32	1.32
VLDL-2	V2CH	5.64	5.64
	V2FC	4.23	4.23
	V2PL	2.00	2.00
	V2TG	1.41	1.41
VLDL-3	V3CH	5.71	5.71
	V3FC	3.62	3.62
	V3PL	2.45	2.45
	V3TG	2.11	2.11
VLDL-4	V4CH	4.73	4.73
	V4FC	5.07	5.07
	V4PL	2.45	2.45
	V4TG	1.06	1.06
VLDL-5	V5CH	0.93	0.93
	V5FC	2.43	2.43
	V5PL	0.77	0.77
	V5TG	0.98	0.98
IDL	IDAB	3.28	3.28
	IDCH	5.27	5.27
	IDFC	5.13	5.13
	IDPL	2.76	2.76
	IDTG	1.48	1.48
LDL	LDAB	1.55	1.55
	LDCH	1.73	1.73
	LDFC	1.03	1.03
	LDPL	1.13	1.13
	LDTG	1.22	1.22
LDL-1	L1AB	1.25	1.25
	L1CH	0.92	0.92
	L1FC	1.14	1.14
	L1PL	0.84	0.84
	L1TG	1.96	1.96
LDL-2	L2AB	9.51	9.51
	L2CH	10.07	10.07
	L2FC	9.80	9.80
	L2PL	7.59	7.59
	L2TG	3.51	3.51
LDL-3	L3AB	1.98	1.98
	L3CH	1.98	1.98
	L3FC	2.39	2.39
	L3PL	1.70	1.70
	L3TG	1.88	1.88

LDL-4	L4AB	1.92	1.92
	L4CH	2.23	2.23
	L4FC	1.87	1.87
	L4PL	2.05	2.05
	L4TG	2.07	2.07
LDL-5	L5AB	2.14	2.14
	L5CH	2.23	2.23
	L5FC	1.92	1.92
	L5PL	1.96	1.96
	L5TG	1.83	1.83
LDL-6	L6AB	1.90	1.90
	L6CH	1.86	1.86
	L6FC	2.05	2.05
	L6PL	1.62	1.62
	L6TG	1.51	1.51
HDL	HDA1	0.51	0.51
	HDA2	0.83	0.83
	HDCH	0.41	0.41
	HDFC	1.25	1.25
	HDPL	0.95	0.95
	HDTG	1.54	1.54
HDL-1	H1A1	2.09	2.09
	H1A2	3.13	3.13
	H1CH	0.58	0.58
	H1FC	2.15	2.15
	H1PL	1.14	1.14
	H1TG	3.93	3.93
HDL-2	H2A1	0.88	0.88
	H2A2	2.63	2.63
	H2CH	1.03	1.03
	H2FC	3.92	3.92
	H2PL	1.07	1.07
	H2TG	2.50	2.50
HDL-3	H3A1	1.03	1.03
	H3A2	2.11	2.11
	H3CH	1.51	1.51
	H3FC	2.08	2.08
	H3PL	1.71	1.71
	H3TG	2.10	2.10
HDL-4	H4A1	0.48	0.48
	H4A2	0.81	0.81
	H4CH	0.70	0.70
	H4FC	1.40	1.40
	H4PL	1.03	1.03
	H4TG	0.94	0.94

TP: Total plasma; VLDL Very-low density lipoprotein; IDL: Intermediate-density lipoprotein; LDL: Low-density lipoprotein; HDL: High-density lipoprotein; A1: Apolipoprotein 1; A2: Apolipoprotein-2; AB: Apolipoprotein-B; CH: Cholesterol; FC: Free cholesterol; PL: Phospholipids; TG: Triglycerides; numbers in name correspond to lipoprotein subfraction.

Figure S1. *Mean raw plasma spectra for different lengths of a centrifugation delay*

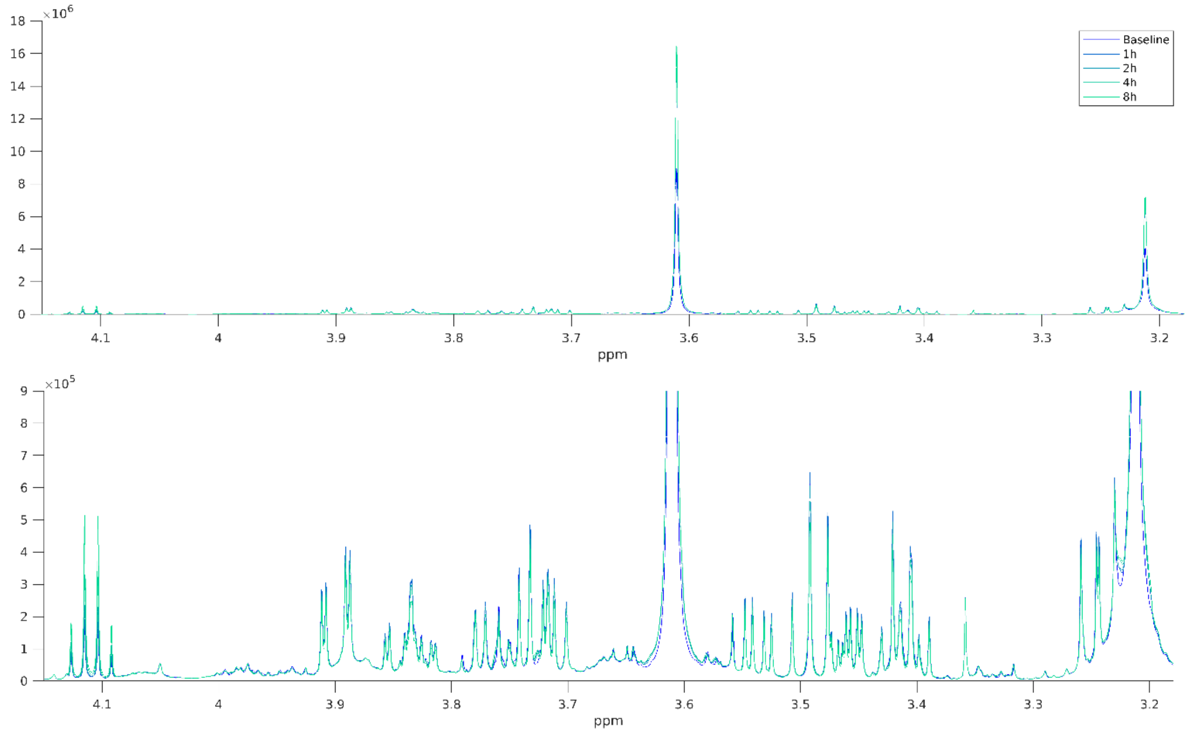

Mean raw plasma spectra, colored according to the length of the centrifugation delay, where the color-scale goes from blue to green, and a lighter color indicates a longer delay.

ppm: parts per million.

__Figure S2.__ PCA loadings plot of plasma metabolites in samples with a different length of centrifugation delay.

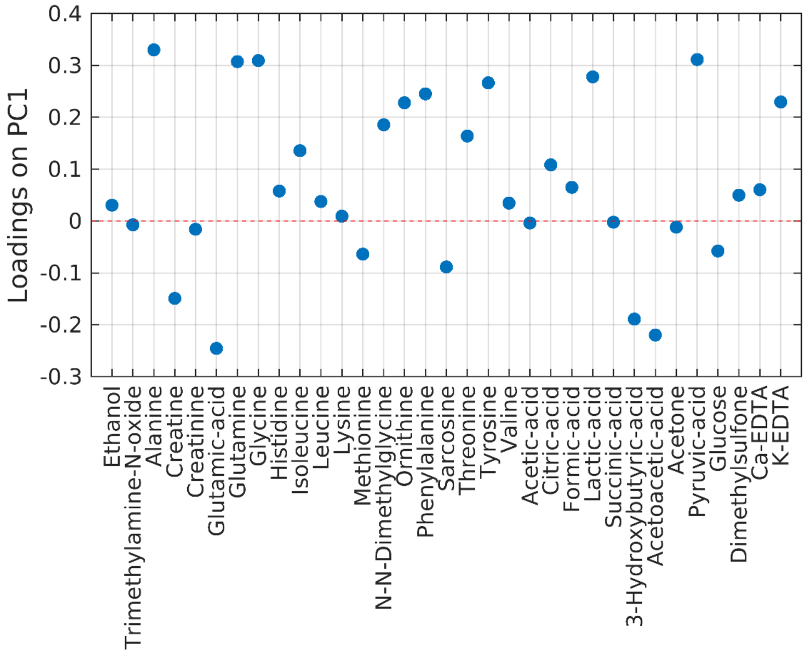

Loading plot corresponding to the PCA scores plot in Figure 2A.

PC1: First principal component

__Figure S3.__ PCA loadings plot of serum metabolites in samples with a different length of centrifugation delay.

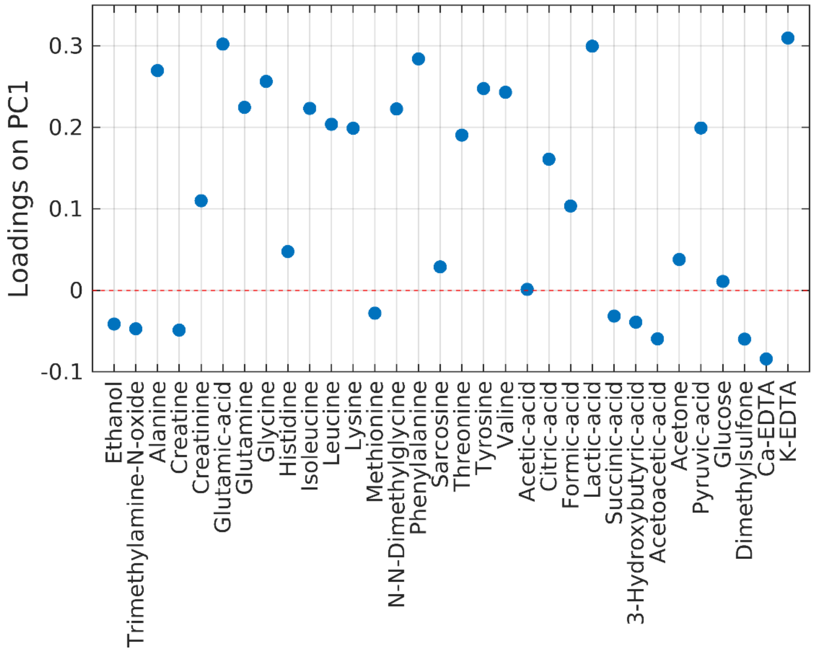

Loading plot corresponding to the PCA scores plot in Figure 2B.

PC1: First principal component

Table 1. *Results from linear mixed models (LMMs) for assessing the significance of changes in metabolite concentrations with a delay in centrifugation (1h-8h).*

	**Plasma**			**Serum**		
**Metabolite**	**Coefficient**	**P-value**	**Q-Value**	**Coefficient**	**P-value**	**Q-value**
Ethanol	0.005 (-0.006, 0.015)	.41	.43	0.001 (0.001, -0.011)	.84	.844
Trimethylamine-N-oxide	0.018 (-0.007, 0.042)	.16	.18	0.020 (0.020, -0.016)	.27	.305
Alanine	0.015 (0.012, 0.018)	<.001	<.001	0.018 (0.018, 0.015)	<.001	<.001
Creatine	0.001 (-0.009, 0.010)	.91	.91	0.015 (0.015, 0.002)	.028	.038
Creatinine	-0.020 (-0.026, -0.013)	<.001	<.001	-0.009 (-0.009, -0.016)	.007	.010
Glutamic-acid	-0.147 (-0.178, -0.115)	<.001	<.001	0.154 (0.154, 0.128)	<.001	<.001
Glutamine	0.024 (0.017, 0.030)	<.001	<.001	0.023 (0.023, 0.017)	<.001	<.001
Glycine	0.007 (0.003, 0.012)	.001	.001	0.039 (0.039, 0.034)	<.001	<.001
Histidine	-0.016 (-0.023, -0.010)	<.001	<.001	0.019 (0.019, 0.012)	<.001	<.001
Isoleucine	0.028 (0.018, 0.038)	<.001	<.001	0.015 (0.015, 0.006)	.002	.002
Leucine	0.011 (0.003, 0.019)	.008	.011	0.024 (0.024, 0.017)	<.001	<.001
Lysine	-0.029 (-0.076, 0.017)	.22	.25	0.007 (0.007, -0.003)	.16	.19
Methionine	-0.141 (-0.161, -0.120)	<.001	<.001	-0.033 (-0.033, -0.040)	<.001	<.001
N-N-Dimethylglycine	0.023 (0.012, 0.033)	<.001	<.001	0.067 (0.067, 0.050)	<.001	<.001
Ornithine	0.115 (0.089, 0.142)	<.001	<.001			
Phenylalanine	0.018 (0.011, 0.026)	<.001	<.001	0.059 (0.059, 0.051)	<.001	<.001
Sarcosine	0.055 (0.037, 0.074)	<.001	<.001	0.003 (0.003, -0.024)	.84	.84
Threonine	-0.045 (-0.093, 0.003)	.064	.082	0.100 (0.100, 0.052)	<.001	<.001
Tyrosine	0.013 (0.009, 0.017)	<.001	<.001	0.007 (0.007, 0.003)	.001	.001
Valine	-0.003 (-0.007, 0.002)	.23	.25	0.009 (0.009, 0.005)	<.001	<.001
Acetic-acid	-0.124 (-0.132, -0.116)	<.001	<.001	-0.040 (-0.040, -0.048)	<.001	<.001
Citric-acid	-0.013 (-0.020, -0.006)	<.001	<.001	0.033 (0.033, 0.026)	<.001	<.001
Formic-acid	-0.001 (-0.016, 0.013)	.85	.88	0.064 (0.064, 0.039)	<.001	<.001
Lactic-acid	0.168 (0.161, 0.175)	<.001	<.001	0.136 (0.136, 0.129)	<.001	<.001
Succinic-acid	0.066 (0.049, 0.083)	<.001	<.001	-0.003 (-0.003, -0.025)	.75	.80
3-Hydroxybutyric-acid	0.016 (-0.006, 0.037)	.15	.18	0.026 (0.026, 0.003)	.030	.038
Acetoacetic-acid	-0.044 (-0.064, -0.024)	<.001	<.001	-0.015 (-0.015, -0.035)	.14	.17
Acetone	0.070 (0.058, 0.082)	<.001	<.001	0.064 (0.064, 0.049)	<.001	<.001
Pyruvic-acid	0.070 (0.063, 0.077)	<.001	<.001	0.022 (0.022, 0.015)	<.001	<.001
Glucose	-0.039 (-0.041, -0.036)	<.001	<.001	-0.032 (-0.032, -0.035)	<.001	<.001
Dimethylsulfone	0.021 (0.013, 0.030)	<.001	<.001	-0.056 (-0.056, -0.070)	<.001	<.001
Ca-EDTA	0.001 (<0.001, 0.002)	.030	.040	-0.013 (-0.013, -0.050)	.50	.55
K-EDTA	0.070 (0.058, 0.082)	<.001	<.001	0.024 (0.024, 0.017)	<.001	<.001

LMM was performed on log-transformed metabolite concentrations, including centrifugation delay as a fixed effect (continuous variable), and individual ID as a random effect. Coefficients are given as coefficient (95% confidence interval). Q-values are multiple testing corrected P-values using the Benjamini-Hochberg procedure.

Table S4. *Mean percentage changes of metabolite levels under a centrifugation delay of 1h, 2h, 4h and 8h, compared to baseline concentrations (centrifugation within 30 minutes)*

	Plasma				Serum			
	Mean percentage change compared to baseline (%)				Mean percentage change compared to baseline (%)			
**Metabolite**	**1h**	**2h**	**4h**	**8h**	**1h**	**2h**	**4h**	**8h**
Ethanol	2.7	19.9	0.6	5.0	5.2	1.5	3.5	0.6
Trimethylamine-N-oxide	15.0	20.7	11.6	23.2	23.5	5.4	31.9	8.9
Alanine	0.3	3.4	9.7	10.4	1.0	4.1	9.6	13.7
Creatine	1.3	-2.0	-9.1	4.9	36.8	31.7	33.3	32.1
Creatinine	-9.3	-8.7	-14.7	-12.0	-7.3	-6.4	-6.5	-5.9
Glutamic-acid	-61.6	-63.5	-64.3	-61.3	28.6	27.3	126.9	211.7
Glutamine	22.3	20.8	21.6	18.8	20.2	21.7	22.9	16.9
Glycine	2.9	2.5	8.0	4.5	4.5	9.0	19.1	32.9
Histidine	-5.6	-9.3	-6.4	-11.7	-1.6	-0.4	8.8	14.8
Isoleucine	13.1	14.0	17.0	24.4	-2.9	-2.5	13.3	10.4
Leucine	0.0	1.9	3.6	9.4	3.7	2.7	13.9	19.4
Lysine	2.2	-8.6	81.1	52.9	6.1	1.8	5.9	7.9
Methionine	-34.3	-36.6	-55.6	-61.4	-16.5	-18.0	-22.0	-20.3
N-N-Dimethylglycine	11.4	14.7	19.7	22.6	54.4	63.6	82.6	84.1
Ornithine	47.0	100.5	113.3	147.2				
Phenylalanine	6.5	6.4	8.3	15.9	5.3	21.7	35.4	52.5
Sarcosine	58.3	62.6	93.8	72.5	60.6	94.7	25.0	9.4
Threonine	24.9	38.5	-17.2	15.2	66.2	76.0	91.1	103.8
Tyrosine	5.6	3.5	8.7	10.1	-0.3	-0.8	1.2	6.3
Valine	-3.8	-0.8	0.5	-3.0	1.2	4.9	4.5	7.2
Acetic-acid	-12.7	-25.2	-39.5	-60.8	-8.2	-13.3	-18.1	-23.6
Citric-acid	-5.8	-4.4	-6.0	-9.6	12.0	13.6	20.6	27.1
Formic-acid	-14.2	-1.6	-2.8	1.9	86.8	89.4	74.0	57.3
Lactic-acid	11.1	48.0	122.8	232.2	22.9	50.7	99.4	164.5
Succinic-acid	-0.4	-4.5	16.2	111.0	59.2	38.0	29.4	20.4
3-Hydroxybutyric-acid	1.6	11.5	10.4	19.4	-5.8	2.3	10.7	23.5
Acetoacetic-acid	12.9	8.4	-13.2	-20.9	-5.2	5.1	-1.0	-12.0
Acetone	14.0	20.5	60.6	65.5	6.5	18.6	38.4	72.2
Pyruvic-acid	-2.0	15.2	42.9	67.1	1.8	-1.4	4.2	22.1
Glucose	-1.4	-4.6	-10.5	-25.3	-1.6	-5.7	-10.0	-21.5
Dimethylsulfone	14.3	20.5	22.6	23.7	-29.5	-25.9	-30.9	-34.8
Ca-EDTA	-0.2	-0.4	1.3	0.5	30.9	37.5	-13.9	28.0
K-EDTA	60.8	64.2	67.6	65.6	9.7	12.8	15.9	20.4

Table S5. *Coefficients of variation (CV) of metabolite concentrations calculated including only the baseline samples (centrifugation within 30 minutes) and including all samples.*

**Metabolite**	**Plasma**		**Serum**	
	**CV 30 min**	**CV all samples**	**CV 30 min**	**CV all samples**
Ethanol	9.9	15.1	14.6	14.5
Trimethylamine-N-oxide	28.6	34.2	48.1	43.6
Alanine	3.4	5.7	3.5	6.6
Creatine	14.2	14.8	11.7	19.2
Creatinine	7.4	10.5	7.0	10.0
Glutamic-acid	14.1	64.7	20.1	51.6
Glutamine	5.6	9.0	3.9	8.8
Glycine	5.0	5.6	5.1	12.5
Histidine	7.5	10.9	10.5	10.8
Isoleucine	14.0	16.0	12.9	15.3
Leucine	10.1	12.4	10.2	11.8
Lysine	24.6	47.5	9.2	11.9
Methionine	10.4	50.4	7.1	13.1
N-N-Dimethylglycine	14.7	18.5	25.7	23.3
Ornithine	29.9	38.6	-	-
Phenylalanine	9.8	10.9	10.4	19.3
Sarcosine	24.9	26.4	41.4	43.1
Threonine	37.1	47.1	60.4	37.9
Tyrosine	6.2	6.1	6.0	6.0
Valine	5.6	6.8	6.6	7.0
Acetic-acid	4.8	34.7	8.7	17.2
Citric-acid	8.8	8.9	9.2	11.0
Formic-acid	17.6	24.6	32.3	30.5
Lactic-acid	5.4	52.4	4.3	39.5
Succinic-acid	24.7	50.2	34.4	45.5
3-Hydroxybutyric-acid	29.1	29.5	30.3	25.6
Acetoacetic-acid	21.4	32.8	25.7	25.7
Acetone	15.7	27.2	16.8	27.7
Pyruvic-acid	4.0	23.1	4.1	12.5
Glucose	1.5	11.8	1.8	9.9
Dimethylsulfone	15.3	11.9	16.3	26.4
Ca-EDTA	0.8	1.3	58.7	62.3
K-EDTA	1.2	19.3	8.3	10.2

Figure S4. *ROC curve for the lactate/glucose ratio*

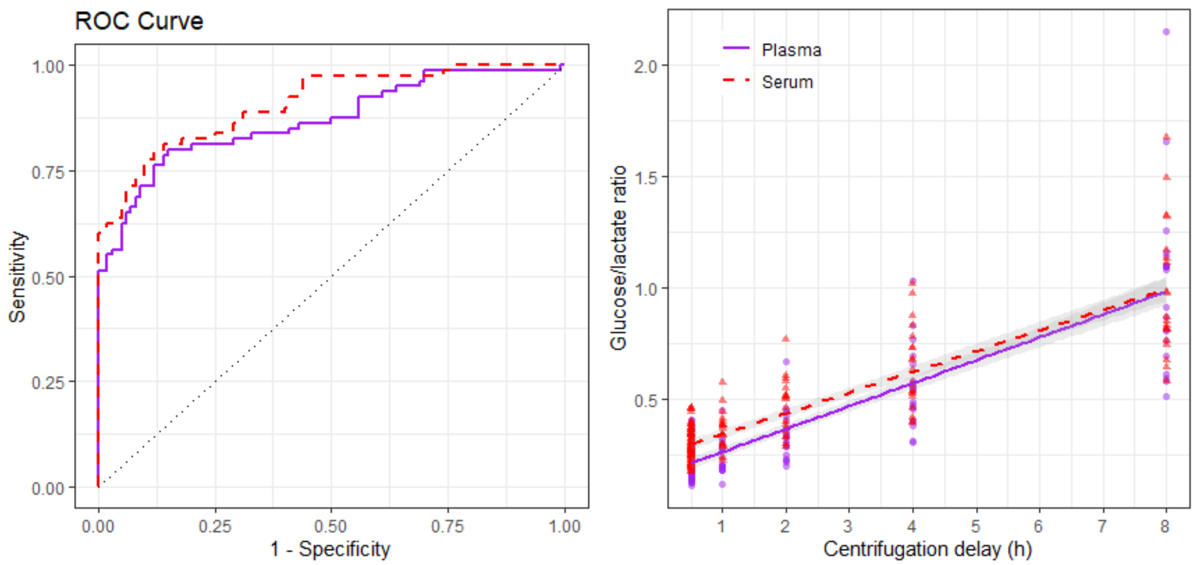

(Left) Receiver operating characteristics curve (ROC) for the lactate/glucose ratio for plasma (solid, purple) and serum (dashed, red) for a centrifugation delay 1h or more. (Right). The lactate/glucose ratio for different centrifugation delays for plasma ans serum samples.

Figure S5. *PCA loadings plot of plasma levels of lipoprotein subfractions in samples with a different length of centrifugation delay.*

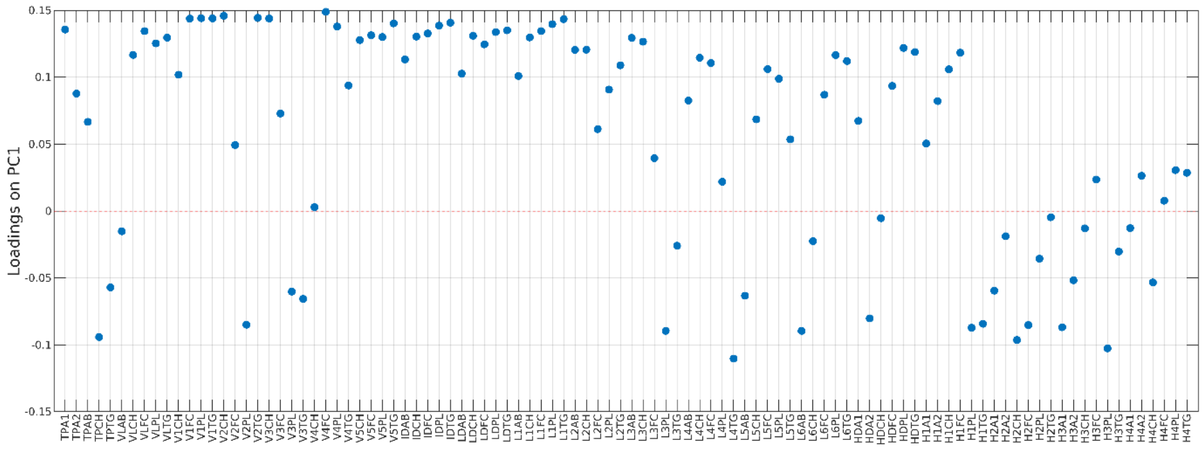

Loading plot corresponding to the PCA scores plot in Figure 4A.

PC1: First principal component; TP: Total plasma; VL: Very-low density lipoprotein; ID: Intermediate-density lipoprotein; LD: Low-density lipoprotein; HD: High-density lipoprotein; A1: Apolipoprotein 1; A2: Apolipoprotein-2; AB: Apolipoprotein-B; CH: Cholesterol; FC: Free cholesterol; PL: Phospholipids; TG: Triglycerides; numbers in name correspond to lipoprotein subfraction.

Figure S6. PCA loadings plot of serum levels of lipoprotein subfractions in samples with a different length of centrifugation delay.

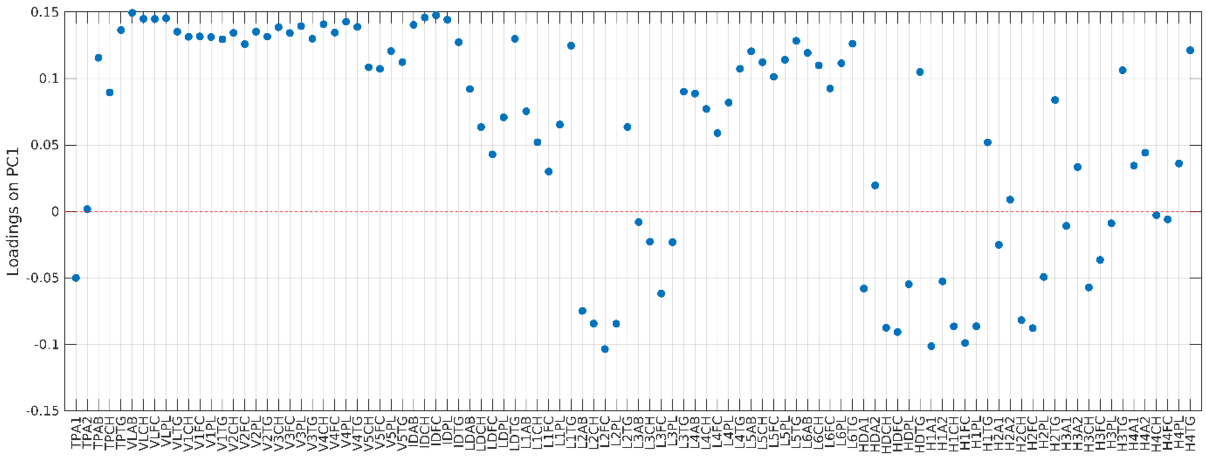

Loading plot corresponding to the PCA scores plot in Figure 2B.

PC1: First principal component; TP: Total plasma; VL: Very-low density lipoprotein; ID: Intermediate-density lipoprotein; LD: Low-density lipoprotein; HD: High-density lipoprotein; A1: Apolipoprotein 1; A2: Apolipoprotein-2; AB: Apolipoprotein-B; CH: Cholesterol; FC: Free cholesterol; PL: Phospholipids; TG: Triglycerides; numbers in name correspond to lipoprotein subfraction.

Table S6. *Results from linear mixed models (LMMs) for assessing the significance of changes in lipoprotein subfraction concentrations with a delay in centrifugation (1h-8h).*

		Plasma			Serum		
**Lipoprotein subfraction**		**Coefficient (95% conf. Int.)**	**P-value**	**Q-value**	**Coefficient (95% conf. Int.)**	**P-value**	**Q-value**
Totals	TPA1	-0,002 (-0,001, -0,003)	<.001	<.001	-0,003 (-0,002, -0,005)	<.001	<.001
	TPA2	0,000 (0,001, -0,002)	.57	.62	0,000 (0,003, -0,002)	.75	.82
	TPAB	0,002 (0,003, 0,000)	.018	.025	-0,002 (0,000, -0,004)	.017	.025
	TPCH	0,001 (0,003, 0,000)	.009	.013	0,000 (0,002, -0,002)	.82	.86
	TPTG	-0,006 (-0,005, -0,007)	<.001	<.001	-0,006 (-0,005, -0,007)	<.001	<.001
VLDL	VLAB	0,001 (0,001, 0,000)	.14	.16	0,001 (0,003, 0,000)	.057	.074
	VLCH	0,009 (0,011, 0,007)	<.001	<.001	0,015 (0,018, 0,013)	<.001	<.001
	VLFC	0,001 (0,002, 0,000)	.11	.13	0,003 (0,005, 0,001)	.002	.003
	VLPL	-0,004 (-0,003, -0,005)	<.001	<.001	-0,002 (0,000, -0,003)	.11	.14
	VLTG	-0,009 (-0,007, -0,011)	<.001	<.001	-0,006 (-0,004, -0,008)	<.001	<.001
VLDL-1	V1CH	0,007 (0,010, 0,005)	<.001	<.001	0,015 (0,019, 0,012)	<.001	<.001
	V1FC	-0,018 (-0,009, -0,027)	<.001	<.001	-0,007 (0,000, -0,014)	.060	.077
	V1PL	-0,008 (-0,005, -0,012)	<.001	<.001	-0,004 (-0,001, -0,007)	.008	.013
	V1TG	-0,010 (-0,006, -0,015)	<.001	<.001	-0,004 (0,000, -0,008)	.039	.051
VLDL-2	V2CH	0,015 (0,020, 0,011)	<.001	<.001	0,019 (0,024, 0,013)	<.001	<.001
	V2FC	0,016 (0,020, 0,012)	<.001	<.001	0,023 (0,032, 0,014)	<.001	<.001
	V2PL	-0,008 (-0,005, -0,011)	<.001	<.001	-0,011 (-0,008, -0,014)	<.001	<.001
	V2TG	-0,024 (-0,019, -0,029)	<.001	<.001	-0,023 (-0,019, -0,027)	<.001	<.001
VLDL-3	V3CH	0,004 (0,021, -0,012)	.61	.65	0,024 (0,032, 0,017)	<.001	<.001
	V3FC	0,009 (0,013, 0,005)	<.001	<.001	0,011 (0,017, 0,006)	<.001	<.001
	V3PL	0,000 (0,003, -0,003)	.79	.79	0,000 (0,004, -0,005)	.88	.91
	V3TG	-0,008 (-0,004, -0,011)	<.001	<.001	-0,011 (-0,006, -0,015)	<.001	<.001
VLDL-4	V4CH	0,013 (0,016, 0,009)	<.001	<.001	0,024 (0,036, 0,012)	<.001	<.001
	V4FC	0,006 (0,010, 0,002)	.004	.007	0,025 (0,030, 0,020)	<.001	<.001
	V4PL	0,005 (0,006, 0,003)	<.001	<.001	0,009 (0,012, 0,007)	<.001	<.001
	V4TG	-0,006 (-0,004, -0,008)	<.001	<.001	-0,007 (-0,005, -0,010)	<.001	<.001
VLDL-5	V5CH	0,004 (0,007, 0,001)	.010	.015	0,008 (0,013, 0,002)	.013	.019
	V5FC	0,023 (0,036, 0,010)	.001	.001	0,019 (0,047, -0,010)	.19	.23
	V5PL	-0,003 (-0,001, -0,005)	.010	.015	0,002 (0,005, -0,002)	.42	.47
	V5TG	-0,007 (-0,005, -0,009)	<.001	<.001	-0,002 (0,000, -0,004)	.066	.084
IDL	IDAB	0,009 (0,012, 0,005)	<.001	<.001	0,013 (0,017, 0,008)	<.001	<.001
	IDCH	0,009 (0,026, -0,008)	.28	.32	0,035 (0,051, 0,020)	<.001	<.001

	IDFC	0,012 (0,022, 0,002)	.024	.033	0,032 (0,046, 0,019)	<.001	<.001
	IDPL	0,001 (0,005, -0,002)	.41	.46	0,000 (0,005, -0,004)	.93	.95
	IDTG	-0,024 (-0,016, -0,031)	<.001	<.001	-0,023 (-0,019, -0,028)	<.001	<.001
LDL	LDAB	0,000 (0,002, -0,001)	.59	.63	-0,004 (-0,002, -0,006)	.001	.001
	LDCH	0,000 (0,001, -0,002)	.69	.71	-0,007 (-0,004, -0,010)	<.001	<.001
	LDFC	-0,006 (-0,005, -0,008)	<.001	<.001	-0,007 (-0,005, -0,009)	<.001	<.001
	LDPL	0,000 (0,001, -0,002)	.44	.481	-0,004 (-0,002, -0,006)	<.001	<.001
	LDTG	0,006 (0,008, 0,003)	<.001	<.001	0,004 (0,006, 0,002)	<.001	.001
LDL-1	L1AB	0,000 (0,002, -0,002)	.88	.88	-0,007 (-0,005, -0,009)	<.001	<.001
	L1CH	0,003 (0,006, 0,001)	.002	.003	-0,004 (-0,002, -0,006)	.001	.001
	L1FC	-0,006 (-0,004, -0,007)	<.001	<.001	-0,013 (-0,011, -0,015)	<.001	<.001
	L1PL	0,002 (0,004, 0,000)	.031	.040	-0,004 (-0,003, -0,006)	<.001	<.001
	L1TG	-0,010 (-0,007, -0,013)	<.001	<.001	-0,019 (-0,015, -0,023)	<.001	<.001
LDL-2	L2AB	-0,003 (0,002, -0,008)	.20	.23	-0,068 (-0,055, -0,081)	<.001	<.001
	L2CH	-0,008 (0,000, -0,016)	.047	.059	-0,096 (-0,073, -0,118)	<.001	<.001
	L2FC	-0,017 (-0,006, -0,028)	.002	.004	-0,159 (-0,111, -0,207)	<.001	<.001
	L2PL	-0,002 (0,003, -0,007)	.43	.48	-0,056 (-0,045, -0,066)	<.001	<.001
	L2TG	0,007 (0,010, 0,004)	<.001	<.001	-0,003 (0,001, -0,006)	.11	.14
LDL-3	L3AB	-0,014 (-0,009, -0,019)	<.001	<.001	-0,012 (-0,002, -0,022)	.023	.032
	L3CH	-0,024 (-0,008, -0,040)	.003	.005	-0,014 (-0,009, -0,020)	<.001	<.001
	L3FC	-0,029 (-0,007, -0,051)	.009	.014	-0,011 (-0,007, -0,016)	<.001	<.001
	L3PL	-0,012 (-0,006, -0,018)	<.001	<.001	-0,011 (0,007, -0,028)	.22	.26
	L3TG	0,002 (0,003, 0,001)	.002	.003	0,001 (0,003, -0,001)	.54	.59
LDL-4	L4AB	-0,007 (-0,001, -0,012)	.022	.031	0,018 (0,023, 0,013)	<.001	<.001
	L4CH	-0,005 (0,000, -0,011)	.046	.058	0,016 (0,021, 0,010)	<.001	<.001
	L4FC	-0,005 (-0,001, -0,010)	.025	.033	0,006 (0,011, 0,001)	.020	.028
	L4PL	-0,004 (0,000, -0,009)	.080	.097	0,016 (0,021, 0,012)	<.001	<.001
	L4TG	0,016 (0,022, 0,010)	<.001	<.001	0,038 (0,043, 0,032)	<.001	<.001
LDL-5	L5AB	0,009 (0,014, 0,005)	<.001	<.001	0,012 (0,018, 0,007)	<.001	<.001
	L5CH	0,008 (0,012, 0,004)	<.001	.001	0,011 (0,017, 0,005)	<.001	.001
	L5FC	0,002 (0,006, -0,003)	.46	.50	0,004 (0,009, -0,002)	.17	.21
	L5PL	0,007 (0,010, 0,003)	<.001	.001	0,009 (0,014, 0,004)	<.001	.001
	L5TG	0,018 (0,022, 0,014)	<.001	<.001	0,035 (0,041, 0,029)	<.001	<.001
LDL-6	L6AB	0,006 (0,010, 0,002)	.002	.003	0,000 (0,005, -0,005)	.94	.95
	L6CH	0,005 (0,009, 0,001)	.018	.025	-0,002 (0,003, -0,007)	.46	.52
	L6FC	-0,009 (-0,004, -0,014)	<.001	.001	-0,020 (-0,014, -0,026)	<.001	<.001

	L6PL	0,003 (0,007, 0,000)	.065	.080	-0,002 (0,002, -0,007)	.32	.37
	L6TG	0,004 (0,006, 0,002)	.001	.002	0,000 (0,003, -0,003)	.80	.85
HDL	HDA1	-0,001 (0,000, -0,002)	.023	.031	-0,005 (-0,003, -0,007)	<.001	<.001
	HDA2	0,000 (0,002, -0,001)	.65	.68	0,001 (0,004, -0,001)	.33	.38
	HDCH	0,002 (0,003, 0,000)	.011	.016	-0,004 (-0,002, -0,005)	<.001	.001
	HDFC	-0,008 (-0,006, -0,011)	<.001	<.001	-0,003 (0,000, -0,005)	.024	.033
	HDPL	-0,002 (-0,001, -0,002)	<.001	<.001	-0,002 (0,000, -0,003)	.017	.025
	HDTG	0,009 (0,011, 0,007)	<.001	<.001	0,007 (0,010, 0,005)	<.001	<.001
HDL-1	H1A1	0,022 (0,028, 0,016)	<.001	<.001	-0,001 (0,006, -0,008)	.76	.82
	H1A2	0,012 (0,017, 0,007)	<.001	<.001	0,017 (0,024, 0,011)	<.001	<.001
	H1CH	0,002 (0,004, 0,000)	.11	.13	-0,007 (-0,003, -0,011)	<.001	<.001
	H1FC	-0,004 (0,002, -0,010)	.23	.26	0,002 (0,007, -0,004)	.51	.57
	H1PL	0,003 (0,005, 0,000)	.031	.040	0,000 (0,003, -0,003)	.10	.10
	H1TG	0,020 (0,024, 0,016)	<.001	<.001	0,010 (0,016, 0,005)	<.001	.001
HDL-2	H2A1	0,001 (0,002, 0,000)	.010	.015	0,002 (0,003, 0,000)	.022	.032
	H2A2	0,000 (0,003, -0,002)	.69	.71	0,004 (0,008, 0,000)	.046	.060
	H2CH	0,015 (0,017, 0,013)	<.001	<.001	0,007 (0,010, 0,004)	<.001	<.001
	H2FC	-0,010 (-0,004, -0,015)	<.001	.001	0,004 (0,010, -0,002)	.18	.22
	H2PL	0,010 (0,012, 0,008)	<.001	<.001	0,007 (0,009, 0,005)	<.001	<.001
	H2TG	0,018 (0,021, 0,015)	<.001	<.001	0,007 (0,013, 0,002)	.006	.010
HDL-3	H3A1	-0,004 (-0,003, -0,005)	<.001	<.001	-0,002 (0,000, -0,003)	.009	.014
	H3A2	-0,003 (-0,002, -0,005)	<.001	.001	0,000 (0,003, -0,003)	.83	.86
	H3CH	-0,004 (-0,002, -0,005)	<.001	<.001	-0,002 (0,000, -0,005)	.032	.044
	H3FC	-0,032 (-0,026, -0,037)	<.001	<.001	-0,025 (-0,019, -0,031)	<.001	<.001
	H3PL	-0,007 (-0,006, -0,009)	<.001	<.001	-0,006 (-0,004, -0,008)	<.001	<.001
	H3TG	0,004 (0,006, 0,002)	<.001	<.001	-0,007 (-0,003, -0,010)	<.001	.001
HDL-4	H4A1	-0,006 (-0,005, -0,008)	<.001	<.001	-0,007 (-0,005, -0,008)	<.001	<.001
	H4A2	-0,006 (-0,004, -0,008)	<.001	<.001	-0,003 (0,000, -0,006)	.024	.033
	H4CH	-0,009 (-0,007, -0,011)	<.001	<.001	-0,009 (-0,007, -0,011)	<.001	<.001
	H4FC	-0,032 (-0,028, -0,037)	<.001	<.001	-0,023 (-0,019, -0,027)	<.001	<.001
	H4PL	-0,011 (-0,009, -0,012)	<.001	<.001	-0,009 (-0,007, -0,010)	<.001	<.001
	H4TG	-0,002 (-0,001, -0,003)	.006	.009	-0,005 (-0,003, -0,006)	<.001	<.001

LMM was performed on log-transformed metabolite concentrations, including centrifugation delay as a fixed effect (continuous variable), and individual ID as a random effect. Coefficients are given as coefficient (95% confidence interval). Q-values are multiple testing corrected P-values using the Benjamini-Hochberg procedure. TP: Total plasma; VLDL: Very-low density lipoprotein; IDL: Intermediate density lipoprotein; LDL: Low-density lipoprotein; HDL: High-density lipoprotein; A1: Apolipoprotein-1; A2: Apolipoprotein-2; CH: Cholesterol; FC: Free cholesterol; PL: Phospholipids; TG: Triglycerides.

Table S7. *Mean percentage changes of levels of lipoprotein subfractios under a centrifugation delay of 1h, 2h, 4h and 8h, compared to baseline concentrations (centrifugation within 30 minutes)*

Lipoprotein subfraction		Plasma				Serum			
		Mean percentage change compared to baseline (%)				Mean percentage change compared to baseline (%)			
		1h	2h	4h	8h	1h	2h	4h	8h
Totals	TPA1	-0.8	-0.8	0.3	-2.1	-1.3	-1.1	-0.1	-2.8
	TPA2	-0.2	-0.1	2.0	-1.0	1.1	1.4	3.8	-0.6
	TPAB	-0.8	-0.2	1.9	0.7	-2.0	-2.0	-0.5	-2.1
	TPCH	-0.3	-0.3	1.7	0.7	-0.5	-0.2	1.4	-0.7
	TPTG	-2.4	-2.1	-3.0	-4.6	-1.6	-2.2	-2.8	-4.4
VLDL	VLAB	0.0	0.5	0.6	0.4	0.7	0.7	1.1	0.9
	VLCH	2.5	3.2	4.8	7.1	4.7	7.3	9.0	11.8
	VLFC	-0.1	1.1	1.1	0.4	1.2	1.4	1.4	2.5
	VLPL	-0.7	-0.3	-0.5	-3.0	1.4	0.8	0.5	-1.2
	VLTG	-4.2	-3.5	-3.4	-6.8	-1.0	-2.1	-1.6	-4.5
VLDL-1	V1CH	0.6	1.5	1.7	6.1	3.6	5.1	6.1	12.7
	V1FC	2.6	-0.5	-1.8	-12.4	-3.2	-3.5	-0.8	-3.7
	V1PL	-4.0	-3.2	-3.6	-6.3	-0.8	-2.0	-0.9	-3.2
	V1TG	-7.8	-4.9	-5.3	-7.8	-2.9	-4.6	-2.5	-2.6
VLDL-2	V2CH	4.6	6.4	10.0	12.5	10.0	13.7	14.1	15.7
	V2FC	4.3	5.0	7.3	13.7	18.0	27.1	15.1	19.7
	V2PL	-1.2	-2.0	-1.2	-6.0	0.6	-0.4	-1.0	-8.1
	V2TG	-6.4	-6.7	-8.5	-15.6	-3.5	-5.2	-8.0	-15.6
VLDL-3	V3CH	46.6	4.6	11.3	5.9	15.4	18.9	23.9	18.1
	V3FC	-0.7	0.8	5.6	6.2	8.4	8.8	11.3	8.0
	V3PL	0.1	-1.2	2.8	-0.9	5.1	4.5	6.2	-1.2
	V3TG	-2.6	-2.6	-1.2	-5.7	1.6	0.6	0.3	-7.8
VLDL-4	V4CH	4.6	5.3	10.7	9.0	20.8	20.6	25.5	22.5
	V4FC	-2.1	0.9	4.5	4.2	5.6	10.6	16.1	19.7
	V4PL	0.8	0.8	3.2	3.2	3.1	4.3	7.2	6.6
	V4TG	-1.5	-1.9	-1.1	-4.9	0.5	-0.8	-0.3	-5.7
VLDL-5	V5CH	4.5	4.1	0.8	4.0	2.0	2.6	1.2	6.7
	V5FC	18.4	21.6	12.9	26.8	5.8	8.8	1.1	19.4
	V5PL	0.8	1.1	-2.4	-1.2	-0.2	-0.2	-2.3	2.3
	V5TG	-2.1	-1.9	-4.8	-4.5	-1.1	-1.7	-3.4	-0.7
IDL	IDAB	-1.0	-1.8	4.7	6.3	1.9	4.1	9.4	9.0
	IDCH	-1.9	-4.5	3.8	7.8	11.7	34.4	25.1	62.4
	IDFC	1.2	-3.4	6.9	9.8	11.7	19.9	30.0	33.6

	IDPL	-0.6	-2.8	3.0	0.4	0.4	1.5	5.6	-1.2
	IDTG	-4.0	-2.7	-10.0	-14.3	-3.3	-5.2	-8.2	-15.3
LDL	LDAB	-0.7	-0.5	1.5	-0.2	-1.4	-1.8	-0.5	-3.1
	LDCH	-0.7	-1.0	1.5	-0.8	-3.3	-3.2	-1.6	-5.5
	LDFC	-3.1	-2.7	-2.0	-5.0	-2.7	-2.8	-2.1	-5.4
	LDPL	-0.6	-0.8	1.0	-0.8	-1.8	-1.7	-0.6	-3.6
	LDTG	-2.3	-0.9	1.6	4.3	-1.2	-0.6	1.2	3.0
LDL-1	L1AB	3.1	2.1	1.5	0.2	-2.2	-1.7	-3.2	-4.7
	L1CH	4.1	2.9	3.1	2.6	-1.2	-0.3	-1.3	-2.9
	L1FC	1.5	0.1	-0.6	-4.5	-3.0	-2.7	-4.5	-9.4
	L1PL	3.2	2.2	1.9	1.6	-1.5	-0.6	-1.9	-3.2
	L1TG	0.0	-1.3	-4.4	-6.3	-4.7	-5.2	-8.7	-11.9
LDL-2	L2AB	7.9	2.8	4.6	-2.8	-22.4	-20.3	-26.5	-34.8
	L2CH	10.4	3.8	5.5	-4.9	-26.3	-23.7	-30.2	-39.2
	L2FC	9.9	1.2	5.6	-11.2	-30.2	-27.1	-33.7	-42.6
	L2PL	7.3	2.5	4.5	-1.7	-19.7	-17.2	-23.0	-30.2
	L2TG	1.6	0.3	2.1	6.5	-4.5	-2.8	-2.6	-1.3
LDL-3	L3AB	-5.8	-6.5	-7.2	-9.1	-9.3	-5.4	-1.4	-10.6
	L3CH	-8.7	-9.3	-9.6	-11.8	-6.4	-4.7	-5.9	-10.2
	L3FC	-9.0	-9.0	-7.5	-10.3	-4.9	-4.5	-5.9	-8.1
	L3PL	-5.2	-6.2	-6.4	-7.8	-9.5	-5.5	2.5	-11.0
	L3TG	-0.3	0.2	-0.6	1.6	-0.9	-1.2	-1.9	1.1
LDL-4	L4AB	-11.2	-8.2	-6.4	-4.3	3.5	5.3	12.7	13.3
	L4CH	-10.0	-7.6	-5.4	-3.6	0.6	3.2	11.7	10.6
	L4FC	-9.3	-6.9	-4.0	-4.2	-3.9	-2.0	4.4	3.2
	L4PL	-8.9	-6.6	-4.7	-2.6	2.6	4.4	11.3	11.8
	L4TG	-8.7	-3.8	1.8	14.7	8.0	11.0	18.5	34.0
LDL-5	L5AB	-3.9	-0.4	6.0	6.5	1.8	2.0	9.9	10.0
	L5CH	-4.2	-1.1	5.1	5.2	1.3	1.3	9.1	8.6
	L5FC	-5.9	-2.3	2.5	0.3	0.0	-0.6	5.0	2.8
	L5PL	-3.8	-1.0	4.1	4.3	0.9	0.9	7.3	6.9
	L5TG	-4.3	0.8	6.8	14.3	13.4	15.0	21.4	30.6
LDL-6	L6AB	1.4	2.2	7.3	3.8	3.5	2.0	4.7	-0.2
	L6CH	1.3	2.2	7.5	3.0	2.9	1.4	5.0	-1.5
	L6FC	-2.5	-1.2	2.0	-7.2	-2.1	-4.6	-3.1	-13.5
	L6PL	1.1	1.8	5.7	1.7	2.7	1.2	3.5	-1.8
	L6TG	-0.1	0.9	4.1	2.2	1.4	0.4	1.6	0.3
HDL	HDA1	-0.1	-0.2	1.1	-1.4	-2.6	-2.3	-1.4	-4.1
	HDA2	0.0	0.3	2.2	-0.3	1.3	1.6	3.8	0.1

	HDCH	0.7	0.4	2.3	0.7	-2.0	-1.8	-0.8	-2.9
	HDFC	-5.8	-5.2	-4.9	-6.1	-3.2	-3.1	-2.5	-2.0
	HDPL	-0.1	-0.5	0.1	-1.4	-0.3	-0.2	0.5	-1.5
	HDTG	-2.2	-0.6	1.8	7.3	-4.9	-3.3	0.3	5.8
HDL-1	H1A1	2.6	0.6	8.6	20.9	-12.8	-9.8	-7.4	1.4
	H1A2	-3.0	-3.8	1.1	11.6	3.6	6.1	9.6	13.8
	H1CH	-0.4	-1.8	0.1	2.1	-4.3	-4.6	-4.4	-4.4
	H1FC	-9.2	-9.0	-5.3	-1.8	-4.3	-3.5	-0.5	1.9
	H1PL	-1.0	-2.3	-0.9	3.2	-3.0	-2.5	-1.4	0.7
	H1TG	-1.6	-0.1	5.4	17.9	-10.8	-7.9	-1.8	9.3
HDL-2	H2A1	-0.2	-0.4	0.9	0.7	0.5	0.6	2.1	0.9
	H2A2	-1.3	-2.1	0.6	0.2	2.5	3.1	5.5	2.1
	H2CH	1.0	0.7	6.2	11.9	-3.7	-1.5	1.7	5.6
	H2FC	-8.2	-8.8	-5.2	-7.3	-2.0	-0.8	2.9	1.9
	H2PL	0.3	-0.1	3.4	7.9	-1.8	-0.3	2.5	5.4
	H2TG	-2.7	-1.2	4.8	14.9	-12.9	-10.0	-3.5	6.3
HDL-3	H3A1	-1.4	-1.5	-0.9	-3.4	0.0	0.0	0.7	-1.9
	H3A2	-2.3	-2.4	-0.7	-2.8	1.2	1.2	3.1	-1.0
	H3CH	-1.4	-1.8	-0.4	-3.3	0.4	0.3	1.4	-2.4
	H3FC	-8.0	-8.0	-8.0	-21.5	-4.6	-6.0	-4.8	-17.3
	H3PL	-0.8	-1.5	-1.6	-5.7	0.5	0.0	0.1	-4.7
	H3TG	-2.6	-1.5	0.9	3.1	-9.2	-8.7	-6.7	-4.4
HDL-4	H4A1	-0.1	0.1	0.0	-5.3	0.1	-0.4	-0.5	-5.4
	H4A2	0.5	0.8	1.1	-4.9	3.3	2.7	3.6	-3.0
	H4CH	0.6	0.5	0.1	-7.1	1.5	0.6	0.0	-7.2
	H4FC	-5.9	-4.2	-7.6	-21.6	1.5	-1.4	-3.2	-16.1
	H4PL	0.2	-0.1	-1.7	-8.1	2.1	1.1	-0.2	-6.6
	H4TG	-0.7	0.4	-0.3	-1.7	-0.3	-0.9	-2.0	-3.4

TP: Total plasma; VLDL Very-low density lipoprotein; IDL: Intermediate-density lipoprotein; LDL: Low-density lipoprotein; HDL: High-density lipoprotein; A1: Apolipoprotein 1; A2: Apolipoprotein-2; AB: Apolipoprotein-B; CH: Cholesterol; FC: Free cholesterol; PL: Phospholipids; TG: Triglycerides; numbers in name correspond to lipoprotein subfraction.

Figure S7. *Percentage changes of serum levels of lipoprotein subfractions.*

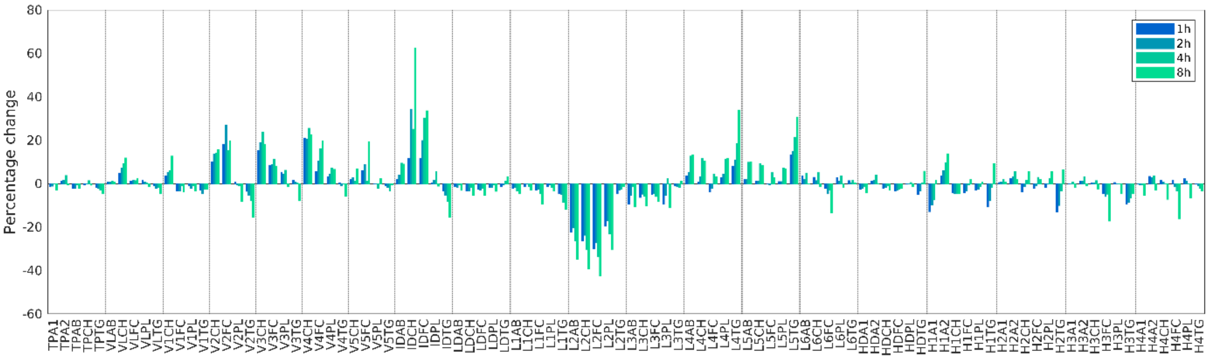

Percentage changes in concentrations of lipoprotein subfractions compared to baseline levels (centrifugation within 30 minutes). The color-scale goes from blue to green, and a lighter color indicates a longer delay.

TP: Total plasma; VL: Very-low density lipoprotein; ID: Intermediate-density lipoprotein; LD: Low-density lipoprotein; HD: High-density lipoprotein; A1: Apolipoprotein 1; A2: Apolipoprotein-2; AB: Apolipoprotein-B; CH: Cholesterol; FC: Free cholesterol; PL: Phospholipids; TG: Triglycerides; numbers in name correspond to lipoprotein subfraction.

Table S8. *Coefficients of variation (CV) of concentrations of lipoprotein subfractions calculated including only the baseline samples (centrifugation within 30 minutes) and including all samples.*

**Lipoprotein subfraction**		**Plasma**		**Serum**	
		**CV 30 min**	**CV all samples**	**CV 30 min**	**CV all samples**
Totals	TPA1	0.76	1.49	1.18	1.80
	TPA2	1.29	2.37	1.88	2.91
	TPAB	1.23	1.92	1.68	2.27
	TPCH	0.85	1.46	1.46	1.79
	TPTG	0.79	1.99	1.24	1.94
VLDL	VLAB	0.74	1.24	1.02	1.39
	VLCH	1.87	3.71	2.50	4.86
	VLFC	1.24	1.44	1.94	2.04
	VLPL	1.17	1.91	1.87	2.31
	VLTG	1.57	3.62	2.11	3.08
VLDL-1	V1CH	2.82	3.75	3.85	5.20
	V1FC	4.96	8.81	5.69	6.97
	V1PL	2.82	4.15	3.19	3.57
	V1TG	3.31	6.03	3.31	5.08
VLDL-2	V2CH	3.03	7.45	3.20	8.53
	V2FC	3.10	7.98	5.06	12.49
	V2PL	1.68	3.66	2.43	5.31
	V2TG	2.50	7.57	3.19	7.37
VLDL-3	V3CH	7.62	17.15	4.92	10.62
	V3FC	3.59	4.87	4.06	6.15
	V3PL	1.97	3.74	2.79	5.59
	V3TG	2.44	4.16	3.13	6.18
VLDL-4	V4CH	2.38	5.46	6.18	7.24
	V4FC	2.91	6.39	4.37	9.07
	V4PL	1.09	2.41	1.61	3.58
	V4TG	1.55	2.95	2.05	4.10
VLDL-5	V5CH	2.91	4.29	4.27	5.38
	V5FC	10.05	12.99	11.39	15.73
	V5PL	1.90	3.07	2.59	4.03
	V5TG	1.22	2.87	1.81	2.70
IDL	IDAB	2.36	5.53	3.82	5.74
	IDCH	4.03	8.83	7.38	12.66
	IDFC	3.80	8.27	7.46	11.16
	IDPL	2.53	4.44	3.58	5.31
	IDTG	3.61	8.61	2.44	7.42
LDL	LDAB	1.29	2.04	1.73	2.62
	LDCH	1.40	2.23	2.07	3.46
	LDFC	1.93	2.86	2.32	3.12
	LDPL	1.07	1.65	1.58	2.40
	LDTG	2.06	3.86	1.94	3.65
LDL-1	L1AB	1.62	2.81	1.59	3.29
	L1CH	2.14	3.01	1.96	3.15
	L1FC	1.44	3.29	2.30	4.52
	L1PL	1.79	2.71	1.57	2.88
	L1TG	1.73	5.48	1.90	6.34
LDL-2	L2AB	5.20	6.39	5.08	20.52
	L2CH	6.47	8.82	6.04	27.91
	L2FC	8.74	11.31	8.88	38.13
	L2PL	4.45	5.83	4.70	16.96
	L2TG	2.28	5.27	2.28	5.33
LDL-3	L3AB	4.37	6.55	6.39	8.65
	L3CH	6.80	12.51	5.88	6.87
	L3FC	6.44	13.59	4.47	5.72
	L3PL	4.18	6.45	7.53	10.33
	L3TG	0.66	1.68	1.23	1.90

LDL-4	L4AB	4.83	8.11	4.75	7.41
	L4CH	4.70	7.57	5.07	7.58
	L4FC	4.61	6.67	4.54	5.88
	L4PL	4.08	6.67	4.01	6.68
	L4TG	4.88	11.00	3.45	12.37
LDL-5	L5AB	3.64	7.02	4.83	7.71
	L5CH	3.45	6.78	4.76	7.62
	L5FC	3.79	6.27	4.35	6.61
	L5PL	3.04	5.78	3.98	6.46
	L5TG	3.60	8.36	4.42	10.99
LDL-6	L6AB	3.11	5.97	4.35	6.89
	L6CH	3.25	6.48	4.37	7.59
	L6FC	3.73	7.61	4.22	9.74
	L6PL	2.61	5.30	3.52	6.21
	L6TG	2.09	3.66	2.83	3.98
HDL	HDA1	0.86	1.50	1.37	2.26
	HDA2	1.23	2.27	1.75	2.76
	HDCH	0.97	1.60	1.50	2.24
	HDFC	2.43	4.16	2.80	3.09
	HDPL	0.67	1.24	1.02	1.42
	HDTG	1.48	4.42	1.69	4.75
HDL-1	H1A1	3.91	9.32	3.69	10.51
	H1A2	3.02	7.85	5.27	7.61
	H1CH	1.81	3.95	2.38	5.09
	H1FC	4.49	8.06	4.37	6.38
	H1PL	1.60	4.10	1.87	4.40
	H1TG	2.76	8.86	2.85	10.14
HDL-2	H2A1	0.72	1.26	1.10	1.57
	H2A2	1.75	3.27	3.01	3.94
	H2CH	1.64	5.43	2.13	4.85
	H2FC	5.04	7.01	5.66	5.36
	H2PL	1.30	3.86	1.54	3.82
	H2TG	2.81	7.50	3.00	9.57
HDL-3	H3A1	0.85	2.07	1.20	1.75
	H3A2	1.56	2.82	2.27	3.28
	H3CH	1.18	2.45	1.72	2.50
	H3FC	4.66	10.71	4.98	9.31
	H3PL	1.09	2.91	1.51	2.95
	H3TG	2.22	3.24	2.33	5.52
HDL-4	H4A1	1.06	2.72	1.39	2.73
	H4A2	1.57	3.67	1.79	3.93
	H4CH	1.41	4.02	1.58	4.07
	H4FC	3.58	9.97	3.65	8.35
	H4PL	1.07	3.85	1.06	3.71
	H4TG	1.36	1.89	1.41	2.27

TP: Total plasma; VLDL Very-low density lipoprotein; IDL: Intermediate-density lipoprotein; LDL: Low-density lipoprotein; HDL: High-density lipoprotein; A1: Apolipoprotein 1; A2: Apolipoprotein-2; AB: Apolipoprotein-B; CH: Cholesterol; FC: Free cholesterol; PL: Phospholipids; TG: Triglycerides; numbers in name correspond to lipoprotein subfraction.

Figure S8. *CVs of lipoprotein subfractions measured in serum samples.*

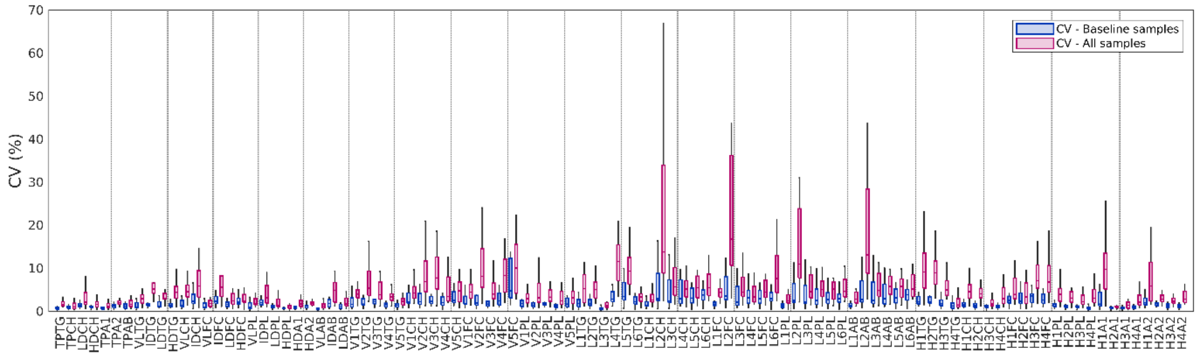

Coefficients of variation (CV) of lipoprotein subfractions measured in serum samples. Blue: Including baseline samples only; Pink: Including samples with a different centrifugation delay.

Total plasma; VL: Very-low density lipoprotein; ID: Intermediate-density lipoprotein; LD: Low-density lipoprotein; HD: High-density lipoprotein; A1: Apolipoprotein 1; A2: Apolipoprotein-2; AB: Apolipoprotein-B; CH: Cholesterol; FC: Free cholesterol; PL: Phospholipids; TG: Triglycerides; numbers in name correspond to lipoprotein subfraction.

Figure S9. *PCA scores plot of serum levels of lipoproteins in healthy vs obese individuals.*

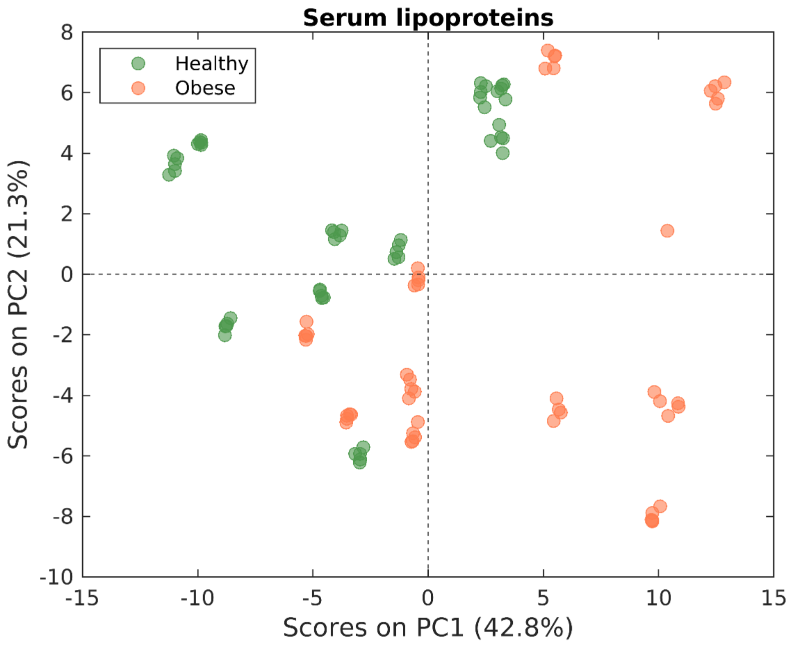

PCA scores plot of levels of serum lipoprotein subfractions in baseline sample aliquots of healthy volunteers (green) and individuals included into the obesity clinic (orange).

PC: Principal component

Figure S10. *PCA loadings plot of serum lipoproteins of healthy vs obese individuals.*

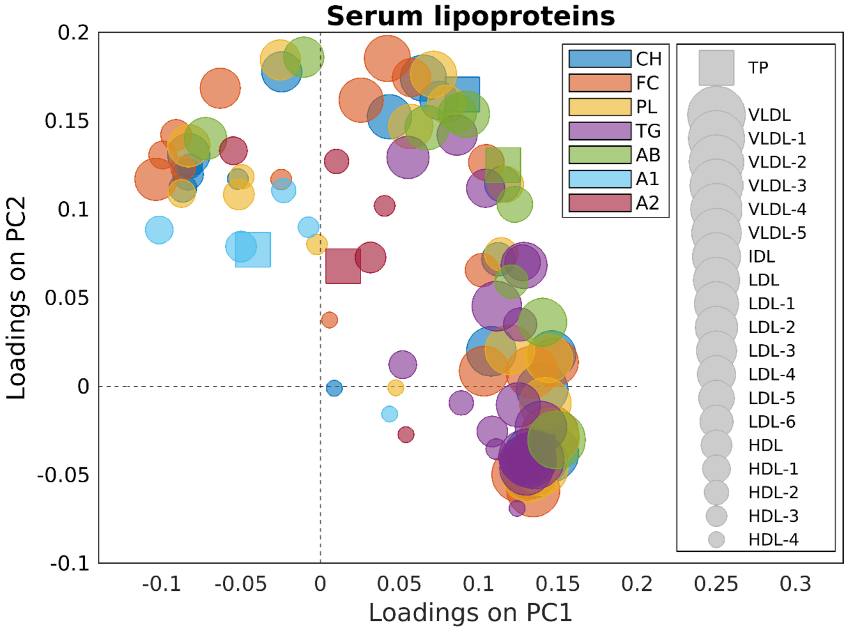

Loading plot corresponding to the PCA scores plot in Figure S9.

PC: Principal component; TP: Total plasma; VLDL Very-low density lipoprotein; IDL: Intermediate-density lipoprotein; LDL: Low-density lipoprotein; HDL: High-density lipoprotein; A1: Apolipoprotein 1; A2: Apolipoprotein-2; AB: Apolipoprotein-B; CH: Cholesterol; FC: Free cholesterol; PL: Phospholipids; TG: Triglycerides; numbers in name correspond to lipoprotein subfraction.

Table S9. *Mean concentrations of lipoprotein subfractions (in mg/dL) between individuals included into the obesity clinic and healthy volunteers.*

**Lipoprotein subfraction**		**Plasma**				**Serum**			
		**Mean conc. obese**	**Mean conc. healthy volunteers**	**P-value**	**Q-value**	**Mean conc. obese**	**Mean conc. healthy volunteers**	**P-value**	**Q-value**
Totals	TPA1	150.7	98.6	<0.001	<0.001	158.2	102.6	<.001	<.001
	TPA2	180.2	182.3	0.40	0.46	178.6	179.7	.49	.55
	TPAB	99.6	104.6	0.21	0.27	96.6	102.0	.29	.35
	TPCH	42.9	55.6	<0.001	<0.001	44.6	57.5	<.001	<.001
	TPTG	121.9	139.5	<0.001	<0.001	123.0	141.0	<.001	<.001
VLDL	VLAB	28.2	30.4	<0.001	<0.001	28.6	30.3	.02	.03
	VLCH	84.4	73.4	0.31	0.37	82.6	71.4	.26	.33
	VLFC	104.8	63.9	<0.001	<0.001	106.8	64.8	<.001	<.001
	VLPL	16.9	8.6	<0.001	<0.001	18.7	9.4	<.001	<.001
	VLTG	18.2	14.0	0.05	0.06	18.4	14.0	.04	.06
VLDL-1	V1CH	10.6	9.1	<0.001	<0.001	10.8	9.1	<.001	.01
	V1FC	23.9	15.1	<0.001	<0.001	23.5	13.8	<.001	<.001
	V1PL	13.5	7.8	<0.001	<0.001	13.4	7.1	<.001	<.001
	V1TG	11.3	7.3	<0.001	<0.001	11.1	6.9	<.001	<.001
VLDL-2	V2CH	3.7	2.2	<0.001	<0.001	3.8	2.0	<.001	<.001
	V2FC	27.2	29.7	0.07	0.10	27.4	30.5	.09	.13
	V2PL	8.6	12.7	<0.001	<0.001	9.1	13.9	<.001	<.001
	V2TG	27.4	17.6	<0.001	<0.001	27.4	17.4	<.001	<.001
VLDL-3	V3CH	7.6	5.0	<0.001	<0.001	8.3	5.4	<.001	<.001
	V3FC	57.4	58.9	0.27	0.33	56.3	57.8	.34	.40
	V3PL	63.1	74.9	<0.001	<0.001	65.3	77.0	<.001	<.001
	V3TG	119.2	139.3	<0.001	<0.001	121.6	141.9	<.001	<.001
VLDL-4	V4CH	29.0	30.6	0.02	0.02	29.2	30.4	.28	.34
	V4FC	9.9	6.4	<0.001	<0.001	10.0	6.4	<.001	<.001
	V4PL	5.2	3.1	<0.001	<0.001	5.6	3.2	<.001	<.001
	V4TG	67.4	63.1	0.73	0.75	65.9	61.5	.85	.86
VLDL-5	V5CH	58.0	33.4	<0.001	<0.001	61.3	35.5	<.001	<.001
	V5FC	17.1	10.4	<0.001	<0.001	17.7	11.0	<.001	<.001
	V5PL	12.6	8.7	<0.001	<0.001	12.6	8.6	<.001	<.001
	V5TG	10.5	7.2	<0.001	<0.001	10.2	6.8	<.001	<.001
IDL	IDAB	3.4	2.8	<0.001	<0.001	3.4	2.7	<.001	<.001
	IDCH	9.3	5.2	<0.001	<0.001	9.3	5.1	<.001	<.001
	IDFC	3.2	2.0	<0.001	<0.001	3.2	1.8	<.001	<.001
	IDPL	3.7	2.2	<0.001	<0.001	3.5	2.0	<.001	<.001

	IDTG	5.3	3.2	<0.001	<0.001	5.3	2.9	<.001	<.001
LDL	LDAB	1.8	1.5	<0.001	<0.001	1.8	1.3	<.001	<.001
	LDCH	4.0	2.0	<0.001	<0.001	4.1	2.1	<.001	<.001
	LDFC	1.4	0.9	<0.001	<0.001	1.3	0.8	<.001	<.001
	LDPL	1.8	1.1	<0.001	<0.001	1.8	1.0	<.001	<.001
	LDTG	2.1	1.3	<0.001	<0.001	2.0	1.2	<.001	<.001
LDL-1	L1AB	0.8	0.5	<0.001	<0.001	0.8	0.4	<.001	<.001
	L1CH	9.8	5.7	<0.001	<0.001	10.1	5.8	<.001	<.001
	L1FC	4.4	2.7	<0.001	<0.001	4.4	2.7	<.001	<.001
	L1PL	4.4	2.9	<0.001	<0.001	4.4	2.8	<.001	<.001
	L1TG	5.3	3.7	<0.001	<0.001	5.2	3.3	<.001	<.001
LDL-2	L2AB	2.3	1.9	<0.001	<0.001	2.2	1.7	<.001	<.001
	L2CH	5.2	3.5	<0.001	<0.001	5.6	3.8	<.001	<.001
	L2FC	1.7	1.8	0.28	0.34	1.9	1.9	.32	.37
	L2PL	2.4	2.1	0.55	0.59	2.4	2.1	.55	.59
	L2TG	2.4	1.8	0.67	0.69	2.3	1.7	.67	.70
LDL-3	L3AB	2.9	1.9	0.03	0.04	2.8	1.7	.01	.02
	L3CH	5.2	3.5	<0.001	<0.001	5.1	3.4	<.001	<.001
	L3FC	17.5	18.1	0.45	0.50	18.4	18.7	.51	.56
	L3PL	8.2	17.0	<0.001	<0.001	10.2	18.6	<.001	<.001
	L3TG	11.0	17.5	<0.001	<0.001	10.8	17.2	<.001	<.001
LDL-4	L4AB	17.0	17.0	0.34	0.40	15.2	15.3	.35	.40
	L4CH	19.9	15.8	0.24	0.30	18.6	14.3	.11	.16
	L4FC	25.9	19.1	<0.001	0.01	23.7	17.3	<.001	<.001
	L4PL	4.9	5.2	0.26	0.32	5.3	5.6	.12	.16
	L4TG	2.2	5.0	<0.001	<0.001	2.8	5.7	<.001	<.001
LDL-5	L5AB	3.0	5.2	<0.001	<0.001	3.1	5.3	<.001	<.001
	L5CH	4.6	4.9	0.21	0.27	4.3	4.7	.26	.33
	L5FC	5.1	4.2	0.43	0.48	4.9	4.1	.45	.51
	L5PL	6.0	4.8	0.02	0.02	5.8	4.7	.08	.11
	L5TG	10.5	10.4	0.96	0.96	11.1	10.9	.88	.88
LDL-6	L6AB	5.2	9.5	<0.001	<0.001	6.1	10.3	<.001	<.001
	L6CH	6.6	9.7	<0.001	<0.001	6.4	9.6	<.001	<.001
	L6FC	9.6	9.4	0.41	0.46	8.7	8.5	.40	.46
	L6PL	10.8	8.7	0.30	0.37	10.3	8.1	.17	.22
	L6TG	14.3	11.1	0.01	0.01	13.4	10.4	.01	.01
HDL	HDA1	9.6	9.0	0.79	0.80	10.2	9.6	.82	.83
	HDA2	5.1	9.0	<0.001	<0.001	6.2	9.9	<.001	<.001
	HDCH	7.0	9.7	<0.001	<0.001	6.9	9.8	<.001	<.001

	HDFC	10.7	9.9	0.66	0.68	9.8	9.1	.65	.69
	HDPL	13.9	10.4	0.11	0.15	13.3	9.6	.07	.10
	HDTG	21.4	15.3	<0.001	<0.001	19.8	13.7	<.001	<.001
HDL-1	H1A1	2.7	2.8	0.57	0.61	2.8	2.9	.65	.69
	H1A2	1.7	1.5	0.02	0.03	1.8	1.5	.02	.02
	H1CH	2.2	1.8	<0.001	<0.001	2.3	1.8	<.001	<.001
	H1FC	4.1	3.2	<0.001	<0.001	4.1	3.1	<.001	<.001
	H1PL	9.9	17.1	<0.001	<0.001	10.8	18.7	<.001	<.001
	H1TG	5.5	8.1	<0.001	<0.001	6.0	8.5	<.001	<.001
HDL-2	H2A1	8.3	9.8	<0.001	<0.001	8.4	10.0	<.001	<.001
	H2A2	19.1	19.9	0.21	0.27	18.8	19.4	.52	.57
	H2CH	2.0	4.5	<0.001	<0.001	2.3	5.0	<.001	<.001
	H2FC	1.2	1.8	<0.001	<0.001	1.3	2.0	<.001	<.001
	H2PL	1.4	1.9	<0.001	<0.001	1.6	2.1	<.001	<.001
	H2TG	3.3	3.4	0.40	0.46	3.3	3.5	.12	.16
HDL-3	H3A1	11.9	20.5	<0.001	<0.001	13.0	22.3	<.001	<.001
	H3A2	9.4	12.2	<0.001	<0.001	10.1	12.8	<.001	<.001
	H3CH	14.0	15.0	0.04	0.06	14.5	15.4	.12	.16
	H3FC	27.4	26.9	0.54	0.59	27.5	26.6	.31	.36
	H3PL	10.2	24.9	<0.001	<0.001	12.3	27.9	<.001	<.001
	H3TG	15.0	17.8	<0.001	<0.001	15.4	18.2	<.001	<.001
HDL-4	H4A1	23.0	25.1	<0.001	<0.001	23.0	25.2	<.001	<.001
	H4A2	73.4	72.4	0.63	0.67	72.2	70.8	.70	.72
	H4CH	1.3	2.6	<0.001	<0.001	1.4	2.7	<.001	<.001
	H4FC	2.8	3.1	0.03	0.05	2.8	3.2	.05	.07
	H4PL	5.7	6.0	0.09	0.13	5.8	6.1	.17	.22
	H4TG	19.1	18.5	0.62	0.65	18.9	17.9	.30	.36

TP: Total plasma; VLDL Very-low density lipoprotein; IDL: Intermediate-density lipoprotein; LDL: Low-density lipoprotein; HDL: High-density lipoprotein; A1: Apolipoprotein 1; A2: Apolipoprotein-2; AB: Apolipoprotein-B; CH: Cholesterol; FC: Free cholesterol; PL: Phospholipids; TG: Triglycerides; numbers in name correspond to lipoprotein subfraction.

Significance assessed using Wilcoxon signed rank tests. Q-values are multiple testing corrected P-values using the Benjamini-Hochberg procedure.

Figure S11. *CVs of plasma levels of lipoprotein subfractions in healthy vs obese individuals.*

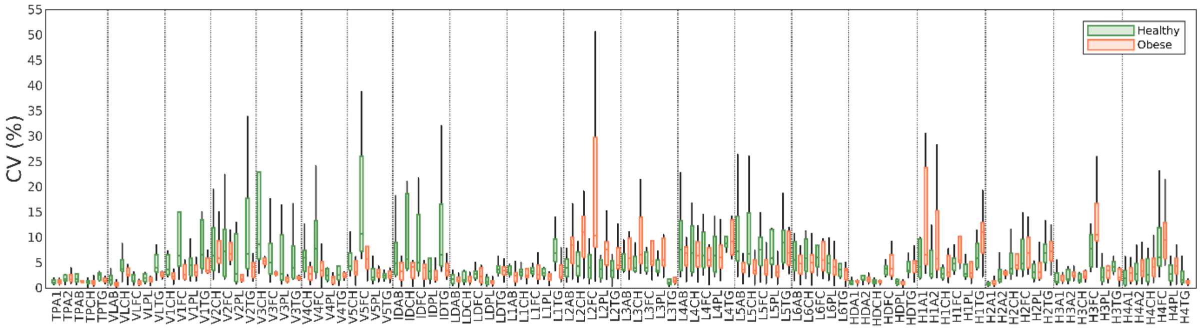

Coefficients of variation (CV) of lipoprotein subfractions measured in plasma samples. Green: Including samples of patients included into the obesity clinic; Orange: Including samples from the healthy volunteers.

TP: Total plasma; VL: Very-low density lipoprotein; ID: Intermediate-density lipoprotein; LD: Low-density lipoprotein; HD: High-density lipoprotein; A1: Apolipoprotein 1; A2: Apolipoprotein-2; AB: Apolipoprotein-B; CH: Cholesterol; FC: Free cholesterol; PL: Phospholipids; TG: Triglycerides; numbers in name correspond to lipoprotein subfraction.

Figure S12. *CVs of serum levels of lipoprotein subfractions in healthy vs obese individuals.*

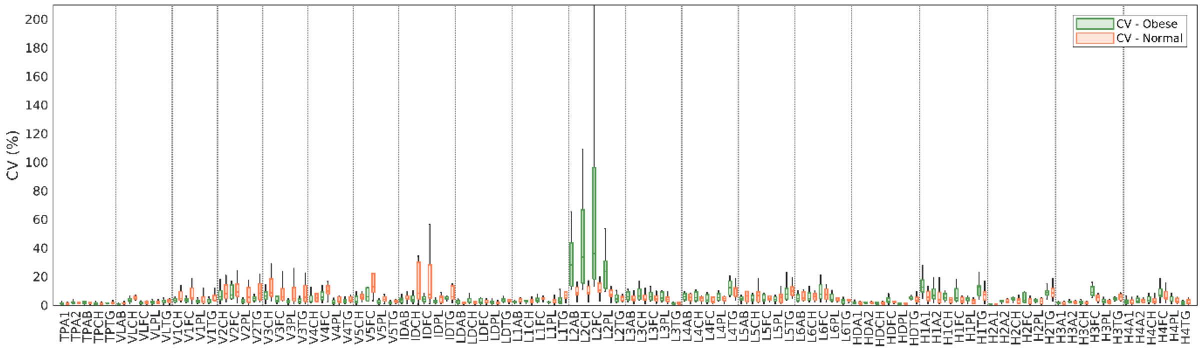

Coefficients of variation (CV) of lipoprotein subfractions measured in serum samples. Green: Including samples of patients included into the obesity clinic; Orange: Including samples from the healthy volunteers.

TP: Total plasma; VL: Very-low density lipoprotein; ID: Intermediate-density lipoprotein; LD: Low-density lipoprotein; HD: High-density lipoprotein; A1: Apolipoprotein 1; A2: Apolipoprotein-2; AB: Apolipoprotein-B; CH: Cholesterol; FC: Free cholesterol; PL: Phospholipids; TG: Triglycerides; numbers in name correspond to lipoprotein subfraction.

Figure S13. *PCA scores plot of plasma metabolites in healthy vs obese individuals.*

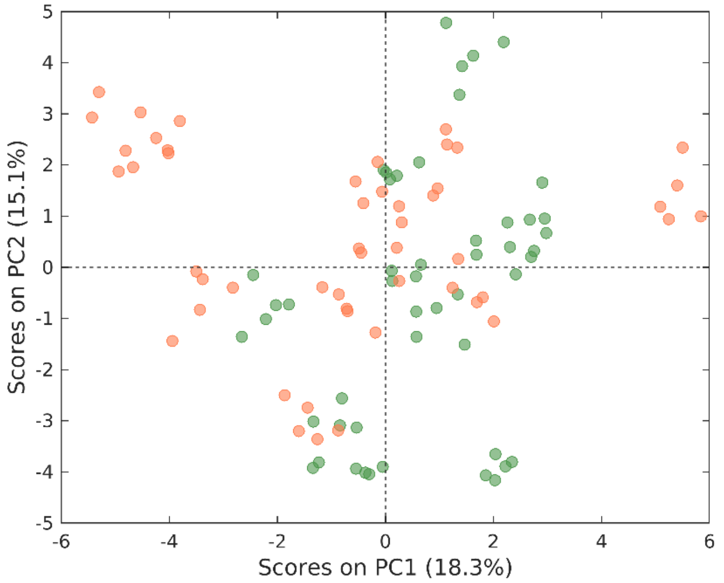

Figure S14. *PCA loadings plot of plasma metabolites in healthy vs obese individuals.*

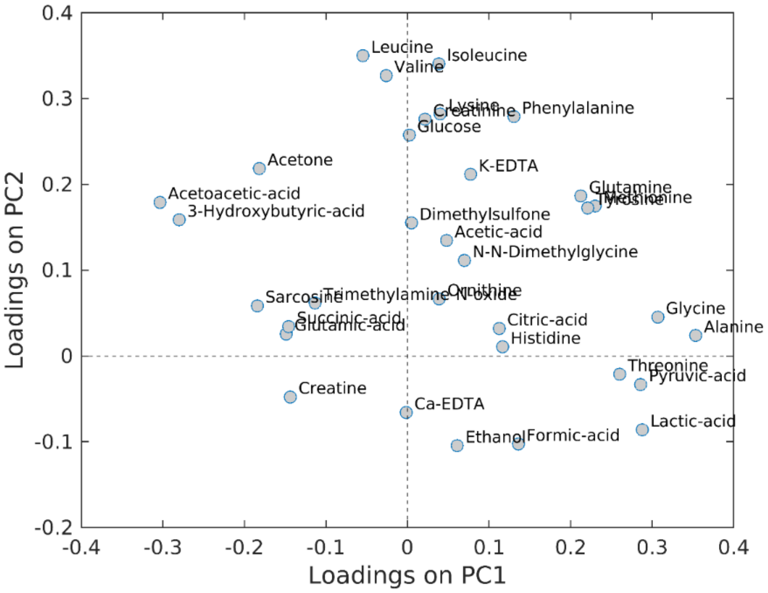

Loadings plot corresponding to the PCA scores plot in Figure 6A.

PC: Principal component

Figure S15. *PCA scores plot of serum metabolites in healthy vs obese individuals.*

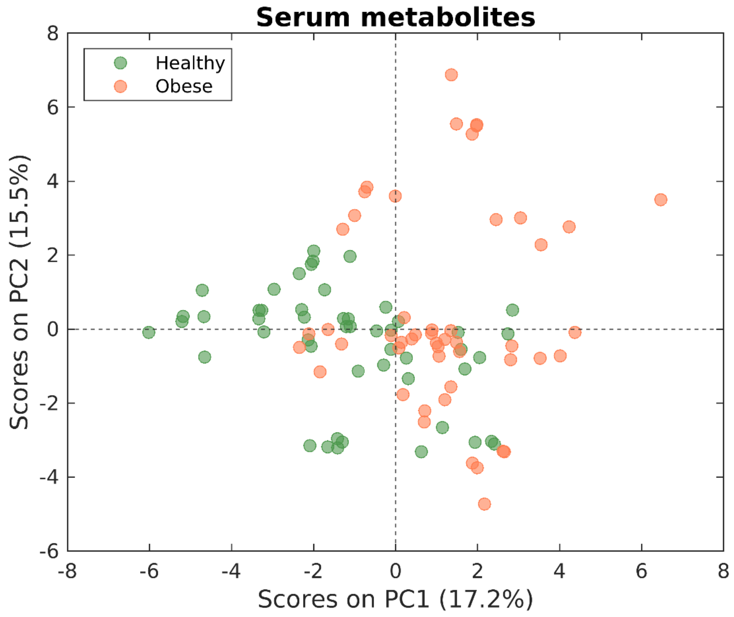

PCA scores plot of serum metabolic levels of baseline aliquots of healthy volunteers (green) and individuals included into the obesity clinic (orange).

PC: Principal component

Figure S16. *PCA loadings plot of serum metabolites in healthy vs obese individuals.*

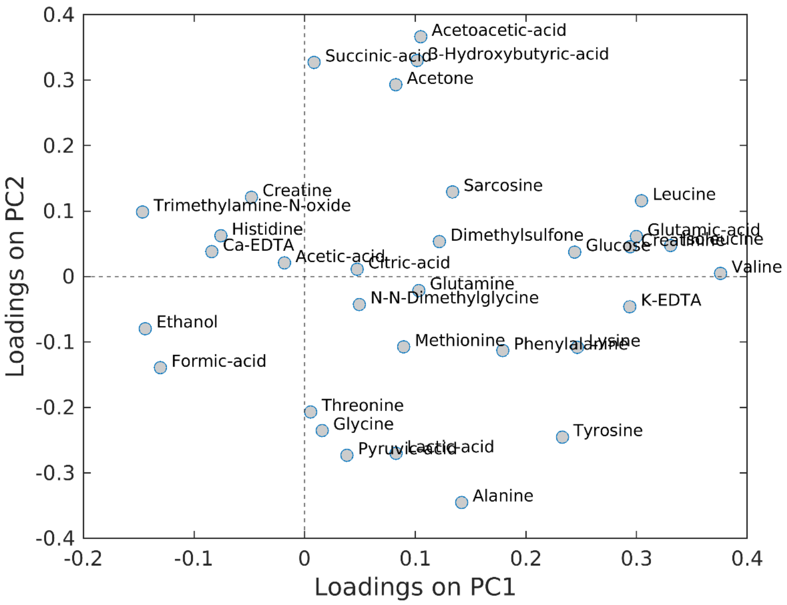

Loadings plot corresponding to the PCA scores plot in Figure S12.

PC: Principal component

Table S10. *Mean metabolite levels (in mmol/L) between individuals included into the obesity clinic and healthy volunteers.*

Metabolite	Plasma				Serum			
	Mean obese	Mean conc. healthy volunteers	P-value	Q-value	Mean obese	Mean conc. healthy volunteers	P-value	Q-value
Ethanol	0.13	0.17	<.001	<.001	0.13	0.18	<.001	<.001
Trimethylamine-N-oxide	0.02	0.03	.01	.02	0.01	0.02	<.001	<.001
Alanine	0.39	0.41	.31	.37	0.43	0.45	.56	.62
Creatine	0.02	0.02	.78	.80	0.02	0.02	.80	.86
Creatinine	0.10	0.07	<.001	<.001	0.10	0.08	<.001	<.001
Glutamic-acid	0.29	0.26	.05	.11	0.11	0.07	<.001	<.001
Glutamine	0.54	0.59	<.001	.01	0.69	0.73	<.001	.01
Glycine	0.22	0.25	<.001	.01	0.23	0.26	<.001	<.001
Histidine	0.07	0.09	<.001	<.001	0.08	0.09	<.001	<.001
Isoleucine	0.06	0.05	<.001	<.001	0.07	0.05	<.001	<.001
Leucine	0.11	0.10	.12	.21	0.12	0.10	.01	.02
Lysine	0.17	0.15	.20	.31	0.21	0.20	.23	.31
Methionine	0.04	0.05	<.001	<.001	0.08	0.09	.02	.04
N-N-Dimethylglycine	0.00	0.01	.01	.02	0.00	0.00	.02	.04
Ornithine	0.02	0.02	.41	.47	-	-		
Phenylalanine	0.04	0.04	.99	.99	0.05	0.05	.18	.26
Sarcosine	0.01	0.01	.31	.37	0.00	0.00	.26	.34
Threonine	0.13	0.15	.27	.35	0.10	0.11	.31	.38
Tyrosine	0.06	0.06	.20	.31	0.06	0.06	.01	.03
Valine	0.28	0.24	<.001	<.001	0.28	0.24	<.001	<.001
Acetic-acid	0.03	0.06	<.001	<.001	0.02	0.05	<.001	<.001
Citric-acid	0.18	0.19	.04	.09	0.15	0.14	.34	.40
Formic-acid	0.03	0.04	.10	.18	0.02	0.02	.01	.02
Lactic-acid	1.21	1.26	.75	.80	1.57	1.57	.93	.93
Succinic-acid	0.00	0.00	.24	.32	0.00	0.00	.06	.11
3-Hydroxybutyric-acid	0.07	0.04	.01	.03	0.09	0.07	.11	.18
Acetoacetic-acid	0.03	0.01	.21	.31	0.02	0.01	.14	.23
Acetone	0.02	0.02	.27	.35	0.02	0.02	.16	.24
Pyruvic-acid	0.15	0.15	.63	.69	0.09	0.10	.16	.24
Glucose	6.15	5.39	<.001	<.001	6.02	5.17	<.001	<.001
Dimethylsulfone	0.00	0.00	.08	.15	0.01	0.01	.90	.93
Ca-EDTA	2.21	2.23	.13	.22	0.00	0.00	.54	.62
K-EDTA	5.88	5.37	<.001	<.001	0.02	0.02	<.001	<.001

Significance assessed using Wilcoxon Signed-Rank tests. Q-values are multiple testing corrected P-values using the Benjamini-Hochberg procedure.

Figure S17. *CVs of plasma metabolites in healthy vs obese individuals.*

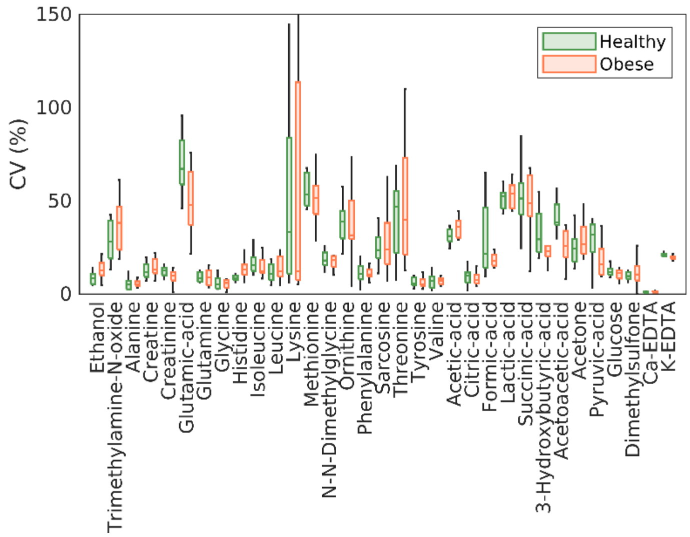

Coefficients of variation (CV) of plasma metabolites. Green: Including samples of patients included into the obesity clinic; Orange: Including samples from the healthy volunteers.

CV: Coefficient of variation

Figure S18. *CVs of serum metabolites in healthy vs obese individuals.*

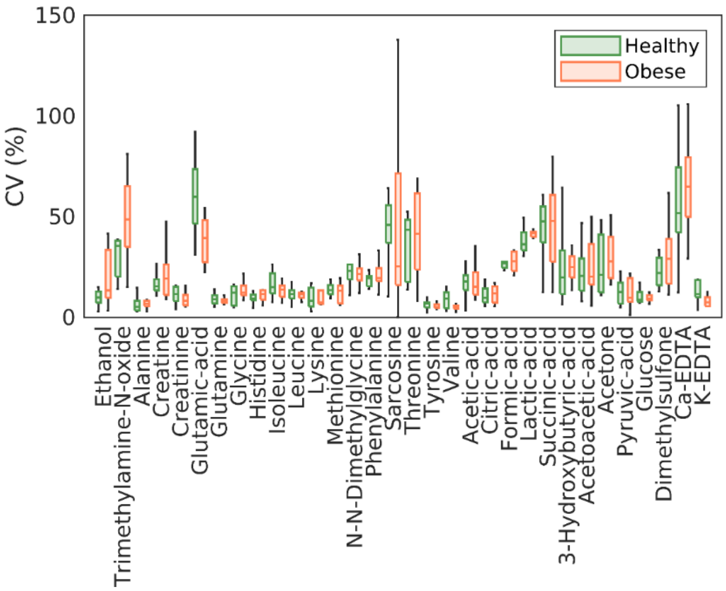

Coefficients of variation (CV) of serum metabolites. Green: Including samples of patients included into the obesity clinic; Orange: Including samples from the healthy volunteers.

CV: Coefficient of variation

Figure S19. *PCA loadings plot of metabolites quantified from plasma vs serum of the same individuals.*

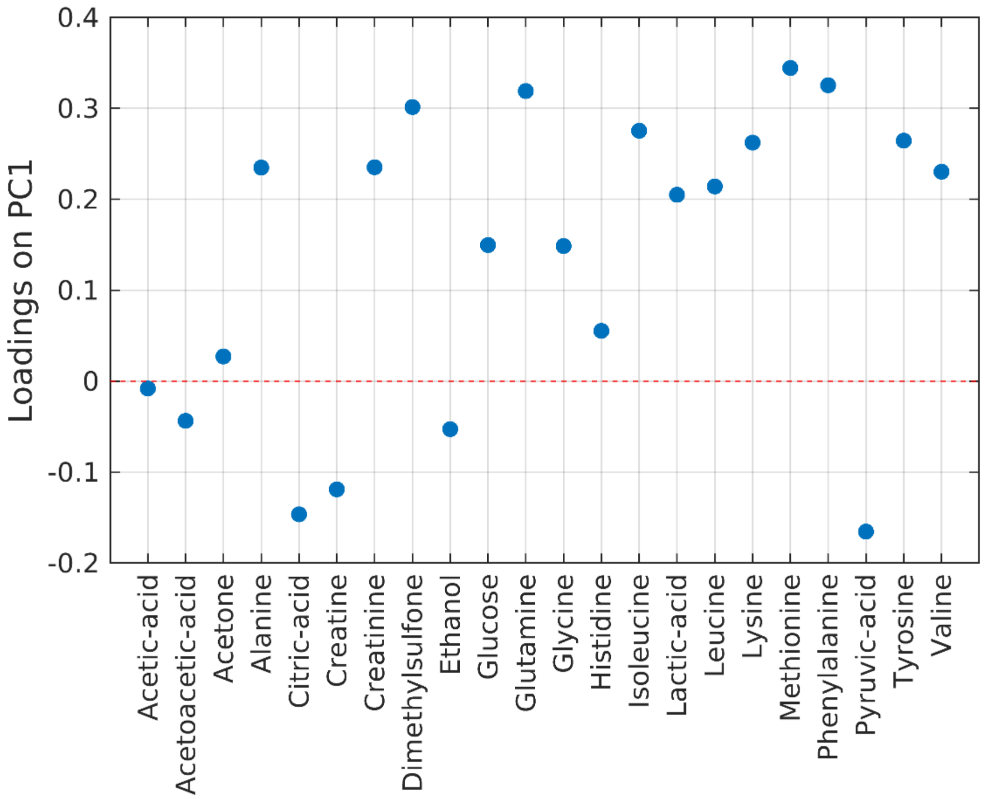

Loadings plot corresponding to the PCA scores plot in Figure 7A.

PC1: First principal component

Table S11. *Differences in concentrations of metabolites quantified from plasma and serum baseline aliquots from the same individuals.*

Metabolite	Plasma		Serum		P-value	Q-value
	Mean conc. (mmol/L)	SD	Mean conc. (mmol/L)	SD		
3-Hydroxybutyric-acid	0.057	0.038	0.082	0.044	<.001	<.001
Acetic-acid	0.047	0.031	0.034	0.027	<.001	<.001
Acetoacetic-acid	0.019	0.021	0.017	0.020	<.001	<.001
Acetone	0.022	0.014	0.022	0.012	.98	.98
Alanine	0.401	0.086	0.438	0.090	<.001	<.001
Ca-EDTA	2.216	0.056	0.003	0.001	<.001	<.001
Citric-acid	0.187	0.021	0.148	0.029	<.001	<.001
Creatine	0.021	0.017	0.019	0.017	<.001	<.001
Creatinine	0.085	0.027	0.090	0.028	<.001	<.001
Dimethylsulfone	0.004	0.003	0.015	0.004	<.001	<.001
Ethanol	0.152	0.043	0.154	0.045	.36	.39
Formic-acid	0.037	0.010	0.019	0.010	<.001	<.001
Glucose	5.770	1.092	5.593	1.090	<.001	<.001
Glutamic-acid	0.276	0.086	0.087	0.036	<.001	<.001
Glutamine	0.566	0.100	0.710	0.088	<.001	<.001
Glycine	0.238	0.059	0.243	0.056	.04	.05
Histidine	0.083	0.013	0.087	0.012	.05	.06
Isoleucine	0.056	0.015	0.060	0.017	.03	.03
K-EDTA	5.626	0.712	0.017	0.003	<.001	<.001
Lactic-acid	1.235	0.287	1.568	0.308	<.001	<.001
Leucine	0.108	0.024	0.111	0.023	.03	.04
Lysine	0.161	0.076	0.205	0.047	<.001	<.001
Methionine	0.043	0.011	0.087	0.016	<.001	<.001
N-N-Dimethylglycine	0.005	0.001	0.004	0.001	<.001	<.001
Phenylalanine	0.042	0.007	0.052	0.007	<.001	<.001
Pyruvic-acid	0.151	0.028	0.092	0.029	<.001	<.001
Sarcosine	0.006	0.004	0.003	0.003	<.001	<.001
Succinic-acid	0.003	0.002	0.002	0.002	<.001	<.001
Threonine	0.142	0.091	0.105	0.055	.01	.01
Trimethylamine-N-oxide	0.024	0.009	0.017	0.009	<.001	<.001
Tyrosine	0.056	0.010	0.060	0.011	<.001	<.001
Valine	0.261	0.039	0.263	0.043	.89	.91

Significance has been tested using paired Wilcoxon Signed-Rank tests, and P-values have been corrected for multiple testing using the Benjamini-Hochberg procedure. SD: Standard deviation

Figure S20. *PCA loadings plot of lipoprotein subfractions measured in plasma vs serum samples of the same individuals.*

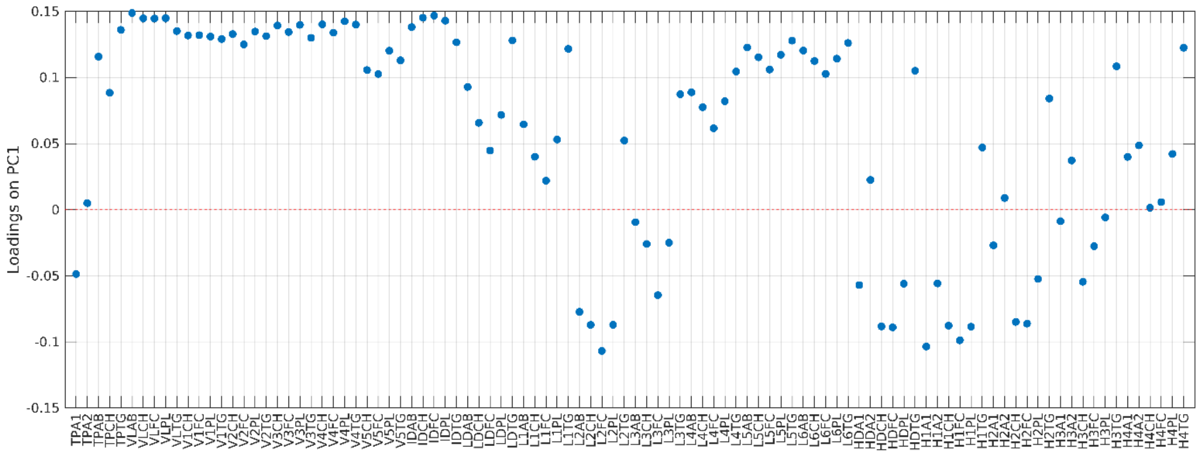

Loadings plot corresponding to the PCA scores plot in Figure 7B.

PC1: First principal component; TP: Total plasma; VL: Very-low density lipoprotein; ID: Intermediate-density lipoprotein; LD: Low-density lipoprotein; HD: High-density lipoprotein; A1: Apolipoprotein 1; A2: Apolipoprotein-2; AB: Apolipoprotein-B; CH: Cholesterol; FC: Free cholesterol; PL: Phospholipids; TG: Triglycerides; numbers in name correspond to lipoprotein subfraction.

Table S12. *Differences in concentrations of lipoprotein subfractions when measured in plasma and serum baseline aliquots from the same individuals.*

Lipoprotein subfraction		Plasma		Serum		P-value	Q-value
		Mean conc. (mg/dL)	SD	Mean conc. (mg/dL)	SD		
Totals	TPA1	130.67	17.62	131.96	18.78	<.001	<.001
	TPA2	29.29	3.54	29.45	3.89	.71	.72
	TPAB	78.94	25.74	77.00	26.37	<.001	<.001
	TPCH	181.28	40.81	179.17	41.94	<.001	<.001
	TPTG	124.69	63.20	130.42	66.88	<.001	<.001
VLDL	VLAB	8.14	3.48	8.18	3.61	.25	.27
	VLCH	19.50	9.66	18.65	10.28	<.001	<.001
	VLFC	9.29	4.29	9.03	4.43	<.001	<.001
	VLPL	22.53	10.36	22.42	10.60	.01	.01
	VLTG	84.33	47.70	85.80	49.58	.02	.02
VLDL-1	V1CH	7.26	4.57	7.20	4.74	<.001	<.001
	V1FC	3.00	2.08	3.10	2.19	<.001	<.001
	V1PL	7.72	4.72	7.99	4.96	<.001	<.001
	V1TG	45.74	29.79	48.36	31.27	<.001	<.001
VLDL-2	V2CH	2.62	1.69	2.53	1.79	<.001	<.001
	V2FC	1.17	0.74	1.05	0.78	<.001	<.001
	V2PL	3.58	2.03	3.58	2.09	.01	.01
	V2TG	13.77	8.75	14.33	8.98	<.001	<.001
VLDL-3	V3CH	2.95	1.65	2.76	1.67	<.001	<.001
	V3FC	1.42	0.82	1.38	0.87	<.001	<.001
	V3PL	3.70	1.75	3.61	1.84	<.001	<.001
	V3TG	10.67	5.75	10.58	6.03	.04	.04
VLDL-4	V4CH	4.23	2.13	4.09	2.33	<.001	<.001
	V4FC	1.71	0.90	1.63	0.87	<.001	<.001
	V4PL	4.49	1.76	4.26	1.90	<.001	<.001
	V4TG	8.85	3.53	8.52	3.76	<.001	<.001
VLDL-5	V5CH	1.68	0.50	1.53	0.55	<.001	<.001
	V5FC	0.64	0.38	0.61	0.39	<.001	<.001
	V5PL	2.08	0.60	1.93	0.62	<.001	<.001
	V5TG	3.12	0.87	3.04	0.87	<.001	<.001
IDL	IDAB	4.13	2.38	4.39	2.51	<.001	<.001
	IDCH	10.63	6.33	10.24	6.74	<.001	<.001
	IDFC	2.96	1.69	2.89	1.83	<.001	<.001
	IDPL	6.30	3.49	6.85	3.81	<.001	<.001
	IDTG	12.79	11.30	14.03	12.24	<.001	<.001
LDL	LDAB	65.23	23.27	63.69	23.74	<.001	<.001
	LDCH	102.10	34.20	99.30	35.09	<.001	<.001
	LDFC	28.41	8.46	28.97	8.83	<.001	<.001
	LDPL	58.17	16.99	57.07	17.44	<.001	<.001
	LDTG	16.10	6.05	16.19	6.30	.80	.80
LDL-1	L1AB	9.32	2.26	9.93	2.47	<.001	<.001
	L1CH	17.80	4.62	18.55	5.08	<.001	<.001
	L1FC	5.05	1.17	5.42	1.24	<.001	<.001
	L1PL	10.47	2.27	10.98	2.48	<.001	<.001
	L1TG	4.37	1.80	4.68	1.94	<.001	<.001
LDL-2	L2AB	7.06	3.14	8.07	3.22	<.001	<.001
	L2CH	12.60	6.75	14.39	6.86	<.001	<.001
	L2FC	3.60	2.01	4.26	2.09	<.001	<.001
	L2PL	7.31	3.32	8.21	3.36	<.001	<.001
	L2TG	1.76	0.57	1.91	0.61	<.001	<.001
LDL-3	L3AB	8.37	3.38	8.36	3.66	.50	.52
	L3CH	14.24	6.63	14.03	6.84	.01	.01
	L3FC	4.10	1.77	4.22	1.78	<.001	<.001
	L3PL	8.12	3.21	8.01	3.45	.02	.02

	L3TG	2.27	0.93	2.30	0.97	<.001	<.001
LDL-4	L4AB	10.30	5.24	9.42	5.44	<.001	<.001
	L4CH	17.03	8.58	15.27	8.94	<.001	<.001
	L4FC	4.76	1.99	4.46	2.07	<.001	<.001
	L4PL	9.50	4.38	8.62	4.57	<.001	<.001
	L4TG	2.10	1.26	2.00	1.31	<.001	<.001
LDL-5	L5AB	12.15	6.42	11.45	6.47	<.001	<.001
	L5CH	17.83	9.36	16.46	9.38	<.001	<.001
	L5FC	4.68	2.14	4.51	2.14	<.001	<.001
	L5PL	9.77	4.67	9.19	4.68	<.001	<.001
	L5TG	2.40	1.45	2.24	1.49	<.001	<.001
LDL-6	L6AB	18.36	8.20	16.75	8.11	<.001	<.001
	L6CH	22.50	10.19	20.50	10.00	<.001	<.001
	L6FC	5.37	2.03	5.27	1.98	<.001	<.001
	L6PL	12.66	4.91	11.91	4.85	<.001	<.001
	L6TG	4.35	2.11	4.26	2.14	<.001	<.001
HDL	HDA1	129.24	19.61	131.77	20.63	<.001	<.001
	HDA2	29.82	3.42	29.83	3.77	.18	.20
	HDCH	49.27	10.03	51.06	10.17	<.001	<.001
	HDFC	10.65	3.01	11.54	3.36	<.001	<.001
	HDPL	69.00	13.13	71.15	13.35	<.001	<.001
	HDTG	9.89	3.03	9.98	3.22	.06	.07
HDL-1	H1A1	17.55	12.06	20.06	12.66	<.001	<.001
	H1A2	1.96	1.19	2.04	1.23	<.001	<.001
	H1CH	13.53	6.18	14.75	6.54	<.001	<.001
	H1FC	3.24	1.78	3.69	1.89	<.001	<.001
	H1PL	16.19	7.74	17.64	8.17	<.001	<.001
	H1TG	2.73	1.05	2.86	1.11	<.001	<.001
HDL-2	H2A1	16.41	3.87	16.79	3.94	<.001	<.001
	H2A2	2.94	1.02	3.01	1.05	<.001	<.001
	H2CH	6.78	2.22	7.23	2.23	<.001	<.001
	H2FC	1.48	0.61	1.67	0.62	<.001	<.001
	H2PL	10.80	3.37	11.43	3.43	<.001	<.001
	H2TG	1.58	0.58	1.66	0.62	<.001	<.001
HDL-3	H3A1	24.07	4.42	24.08	4.58	.39	.41
	H3A2	5.85	1.25	5.97	1.27	<.001	<.001
	H3CH	9.06	1.69	9.18	1.73	<.001	<.001
	H3FC	1.67	0.57	1.84	0.59	<.001	<.001
	H3PL	14.51	3.17	14.93	3.24	<.001	<.001
	H3TG	2.00	0.73	2.05	0.77	<.001	<.001
HDL-4	H4A1	72.91	10.62	71.53	12.02	<.001	<.001
	H4A2	18.81	3.01	18.41	3.49	<.001	<.001
	H4CH	19.49	3.04	19.10	3.40	<.001	<.001
	H4FC	3.35	0.83	3.38	0.94	.40	.42
	H4PL	27.17	3.79	27.03	4.15	.01	.01
	H4TG	3.63	1.13	3.64	1.20	.69	.70

Significance has been tested using paired Wilcoxon signed-rank tests, and P-values have been corrected for multiple testing using the Benjamini-Hochberg procedure. SD: Standard deviation. SD: Standard deviation; TP: Total plasma; VLDL: Very-low density lipoprotein; IDL: Intermediate density lipoprotein; LDL: Low-density lipoprotein; HDL: High-density lipoprotein; A1: Apolipoprotein-1; A2: Apolipoprotein-2; CH: Cholesterol; FC: Free cholesterol; PL: Phospholipids; TG: Triglyceride

Figure S21. *Baseline distortion of serum spectra.*

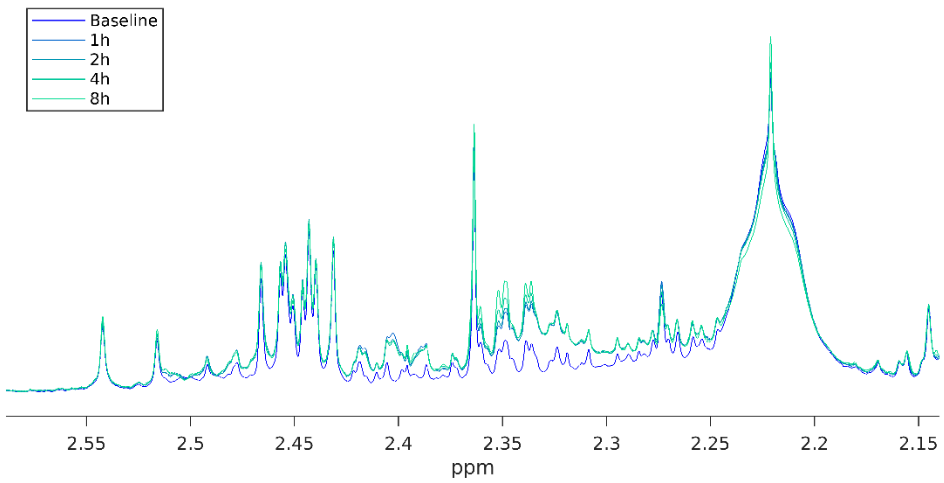

Mean raw plasma spectra, colored according to the length of the centrifugation delay, where the color-scale goes from blue to green, and a lighter color indicates a longer delay.

ppm: parts per million
